# Salmonella Induces the cGAS-STING-Dependent Type I Interferon Response in Murine Macrophages by Triggering mtDNA Release

**DOI:** 10.1128/mbio.03632-21

**Published:** 2022-05-23

**Authors:** Lei Xu, Mengyuan Li, Yadong Yang, Chen Zhang, Zhen Xie, Jingjing Tang, Zhenkun Shi, Shukun Chen, Guangzhe Li, Yanchao Gu, Xiao Wang, Fuhua Zhang, Yao Wang, Xihui Shen

**Affiliations:** a Shaanxi Key Laboratory of Agricultural and Environmental Microbiology, College of Life Sciences, Northwest A&F University, Yangling, Shaanxi, China; School of Medicine, Oregon Health and Science University

**Keywords:** *Salmonella*, cGAS, STING, type I interferon, mtDNA, interferon

## Abstract

Salmonella enterica serovar Typhimurium (*S.* Typhimurium) elicited strong innate immune responses in macrophages. To activate innate immunity, pattern recognition receptors (PRRs) in host cells can recognize highly conserved pathogen-associated molecular patterns (PAMPs). Here, we showed that *S.* Typhimurium induced a robust type I interferon (IFN) response in murine macrophages. Exposure of macrophages to *S.* Typhimurium activated a Toll-like receptor 4 (TLR4)-dependent type I IFN response. Next, we showed that type I IFN and IFN-stimulated genes (ISGs) were elicited in a TBK1-IFN-dependent manner. Furthermore, cytosolic DNA sensor cyclic GMP-AMP synthase (cGAS) and immune adaptor protein stimulator of interferon genes (STING) were also required for the induction of type I IFN response during infection. Intriguingly, *S.* Typhimurium infection triggered mitochondrial DNA (mtDNA) release into the cytosol to activate the type I IFN response. In addition, we also showed that bacterial DNA was enriched in cGAS during infection, which may contribute to cGAS activation. Finally, we showed that cGAS and STING deficient mice and cells were more susceptible to *S.* Typhimurium infection, signifying the critical role of the cGAS-STING pathway in host defense against *S.* Typhimurium infection. In conclusion, in addition to TLR4-dependent innate immune response, we demonstrated that *S.* Typhimurium induced the type I IFN response in a cGAS-STING-dependent manner and the *S.* Typhimurium-induced mtDNA release was important for the induction of type I IFN. This study elucidated a new mechanism by which bacterial pathogen activated the cGAS-STING pathway and also characterized the important role of cGAS-STING during *S.* Typhimurium infection.

## INTRODUCTION

Salmonella enterica serovar Typhimurium (*S.* Typhimurium) is a Gram-negative, intracellular bacterial pathogen. It is recognized as one of the most identified bacterial causes of foodborne illness worldwide (1). *S.* Typhimurium is also an important model organism that is widely employed to investigate the molecular mechanism in bacterial pathogenesis and pathogen-host interactions. *S.* Typhimurium can infect different types of cells, including epithelial cells and macrophages, and can survive within them (2). In macrophages, *S.* Typhimurium infection triggers strong innate immune responses that govern the outcome of infection.

Host innate immune responses to *S.* Typhimurium have drawn great attention. The activation of the innate immune system is featured with proinflammatory transcriptional responses, which are mediated by different pattern recognition receptors (PRRs) that sense a variety of pathogen-associated molecular patterns (PAMPs), like bacterial nucleic acid, cell wall components, or flagellin. Host cells can recognize *S.* Typhimurium lipopolysaccharide (LPS) by a surface innate immune receptor Toll-like receptor 4 (TLR4), which leads to the production of proinflammatory cytokines (2, 3). Accordingly, the mice that are defected in this pathway are more susceptible to *S.* Typhimurium infection, suggesting the sensing of essential PAMPs is vital for protection against *S.* Typhimurium infection (4). Additionally, TLR5 recognizes *S.* Typhimurium flagellin to induce cytokine and chemokine productions (4). The NOD-like receptors (NLRs) that monitor the host cell cytosol are also involved during *S.* Typhimurium infection (4). Two NLRs, NLRP3 (LRR and pyrin domain-containing 3) and NLRC4 (NOD-, LRR- and CARD-containing 4) can sense the infection of *S.* Typhimurium, which cooperatively orchestrates the protective innate immune response (5, 6). The NLRC4 is activated by *S.* Typhimurium flagellin (7) and type III secretion apparatus (T3SS) which is encoded on Salmonella pathogenicity island 1 (SPI-1) (8, 9), while *S.* Typhimurium mRNA is the possible ligand of NLRP3 (10). In addition, *S.* Typhimurium-secreted effectors can act as “danger-associated molecular patterns” that activate Rho-family GTPases through innate immune receptor NOD1, which leads to NF-κB activation (11).

Bacterial-derived cyclic dinucleotides (CDNs) like cyclic di-guanylate monophosphate (c-di-GMP) or cyclic di-AMP (c-di-AMP) can directly bind to STING to induce the production of type I interferons (IFNs) and transcription of IFN-stimulated genes (ISGs) (12–14). In addition, the stimulator of interferon genes (STING) can also be activated by cytosolic DNA sensor cyclic-GMP-AMP synthase (cGAS) (15, 16) which could detect the cytosolic DNA oriented from DNA viruses, bacteria, and mitochondrial DNA (17–20). Although the role of the cGAS-STING pathway in favoring antiviral innate immune response has been extensively investigated (21), its role during bacterial infection is not fully understood. Accumulating studies demonstrated that the cGAS-STING pathway, which is the vital cytosolic surveillance pathway (CSP), is essential for the induction of type I IFN response during the infection of a variety of intracellular bacterial pathogens (13, 19, 22–31). For instance, Mycobacterium tuberculosis, Francisella novicida, and Chlamydia trachomatis engage cGAS via bacterial dsDNA (19, 25, 26), whereas Listeria monocytogenes secreted cyclic di-AMP directly activates STING (13). One important feature of the innate immune response against *S.* Typhimurium infection is the induction of type I interferons (IFNs), including IFN-α and IFN-β (32). Although it has been reported that *S.* Typhimurium induces the type I IFN response through TLRs in host cells (33), whether other CSPs like the cGAS-STING pathway contribute to the induction of type I IFN response is largely unknown.

In this study, by using transcriptomic analysis, we found that type I IFN and many ISGs were potently induced during *S.* Typhimurium infection in murine macrophages. Consistent with a previous study (2), we also demonstrated that *S.* Typhimurium activated a TLR4-dependent pathway. However, we further showed that such type I IFN response could also be induced in a cGAS-STING-dependent fashion. Interestingly, the cGAS-STING-dependent type I IFN response was triggered by the release of mtDNA into the cytosol. Released mtDNA and *S.* Typhimurium DNA in the cytosol activated the cytosolic DNA sensor cGAS. In addition, the lack of cGAS or STING led to significantly increased susceptibility to *S.* Typhimurium infection in the *in vitro* and *in vivo* model, suggesting the critical role of cGAS-STING in supporting host defense against *S.* Typhimurium infection. In conclusion, we demonstrated that *S.* Typhimurium induced the type I IFN response in a cGAS-STING-dependent manner by triggering mtDNA release.

## RESULTS

### *S.* Typhimurium induces proinflammatory cytokines expression during macrophage infection.

**FIG 1:**
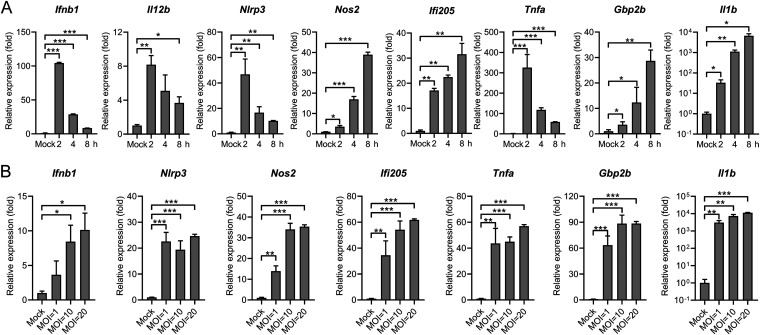
*S.* Typhimurium infection triggers proinflammatory cytokine expression in murine macrophages. (A) qRT-PCR analysis of gene expression in mouse peritoneal macrophages (PMs) uninfected (mock) or infected with *S.* Typhimurium for 2, 4, or 8 h at an MOI of 10 (*n* = 3). (B) qRT-PCR analysis of gene expression in mouse PMs uninfected (mock) or infected with *S.* Typhimurium for 8 h at different MOIs (*n* = 3). Data in (A) and (B) were normalized to uninfected control (mock, set as 1), *Actin* was used as the housekeeping gene. Error bars represent ± SEM. *, *P* < 0.05; **, *P* < 0.01; ***, *P* < 0.001.

Microbial infections elicited strong host innate immune responses through the sensing of conserved microbial products. Proinflammatory cytokines and chemokines have been shown to play essential roles in controlling bacterial infections. To explore the nature of the macrophage innate immune response during *S.* Typhimurium infection, mouse peritoneal macrophages (PMs) were infected with *S.* Typhimurium at different time points. Total RNA from infected cells or uninfected (mock) was harvested after 2 h, 4 h, or 8 h infection since these time points are used as key induction time points of innate immune activation during different intracellular bacterial infections (18–20). The samples were subjected to RT-qPCR to detect the gene expression of diverse proinflammatory cytokines and chemokines. First, cell viability was slightly decreased at 8 h postinfection (8 h.p.i.) (Fig. S1A). Next, we revealed that at the early time point after infection (2 h.p.i.), robust induction of the cytokines, including *Ifnb1*, *Tnfa*, *Il12b*, and inflammasome genes *Nlrp3* was obtained (Fig. 1A), which is consistent with a previous study (34). In contrast, gene expression of two antimicrobial genes *Nos2* and *Gbp2b* (35–37), one *S.* Typhimurium fimbrial protein FimH induced gene *Il1b* (38), and one IFN-activated gene *Ifi205* were peaked at 8 h.p.i (Fig. 1A). Similar gene expression patterns were also confirmed in *S.* Typhimurium-infected murine macrophage Raw264.7 cells (Fig. S1B). To further explore the *S.* Typhimurium-induced proinflammatory cytokine expression, PMs were infected with *S.* Typhimurium at different multiplicity of infections (MOIs). While clear cell death was observed in PMs infected with *S.* Typhimurium at an MOI of 100 (Fig. S1C), all those above-mentioned genes were induced at an MOI of 1 and peaked at an MOI of 10 and 20, with no difference was observed between cells infected at an MOI of 10 or 20 (Fig. 1B). Consistently, significant induction of these genes was also detected in Raw264.7 cells infected at an MOI of 10 (Fig. S1D). Together, these results demonstrated that the *S.* Typhimurium infection triggered proinflammatory cytokine expression in murine macrophages.

10.1128/mbio.03632-21.1 FIG S1 *S.* Typhimurium infection triggers proinflammatory cytokine expression in murine macrophages. (A) CCK8 analysis of cell viability in mouse peritoneal macrophages (PMs) uninfected (mock) or infected with *S.* Typhimurium for 2, 4, or 8 h at an MOI of 10 (*n* = 3). (B) qRT-PCR analysis of gene expression in Raw264.7 cells uninfected (mock) or infected with *S.* Typhimurium for 2, 4, or 8 h at an MOI of 100 (*n* = 3). (C) CCK8 analysis of cell viability in mouse peritoneal macrophages (PMs) uninfected (mock) or infected with *S.* Typhimurium for 8 h at different MOIs (*n* = 3). (D) qRT-PCR analysis of gene expression in Raw264.7 cells uninfected (mock) or infected with *S.* Typhimurium for 8 h at different MOIs (*n* = 3). (E) KEGG pathway analysis results for comparison of upregulated genes in PMs uninfected (mock) or infected with *S.* Typhimurium for 8 h at an MOI of 10. (F) Immunoblot analysis of protein expression in PMs uninfected (mock) or infected with *S*. Typhimurium for 2, 4, or 8 h at an MOI of 10. (G) Immunoblot analysis of protein expression in Raw264.7 cells uninfected (mock) or infected with *S.* Typhimurium for 8 h at different MOIs. Data in (A to D) were normalized to uninfected control (mock, set as 1). *Actin* was used as the housekeeping gene. Error bars represent ± SEM. *P < 0.05; **P < 0.01; ***P < 0.001. Download FIG S1, TIF file, 1.9 MB. Copyright © 2022 Xu et al. 2022 Xu et al. https://creativecommons.org/licenses/by/4.0/ This content is distributed under the terms of the Creative Commons Attribution 4.0 International license.

### *S.* Typhimurium infection triggers type I IFN response in murine macrophages.

**FIG 2:**
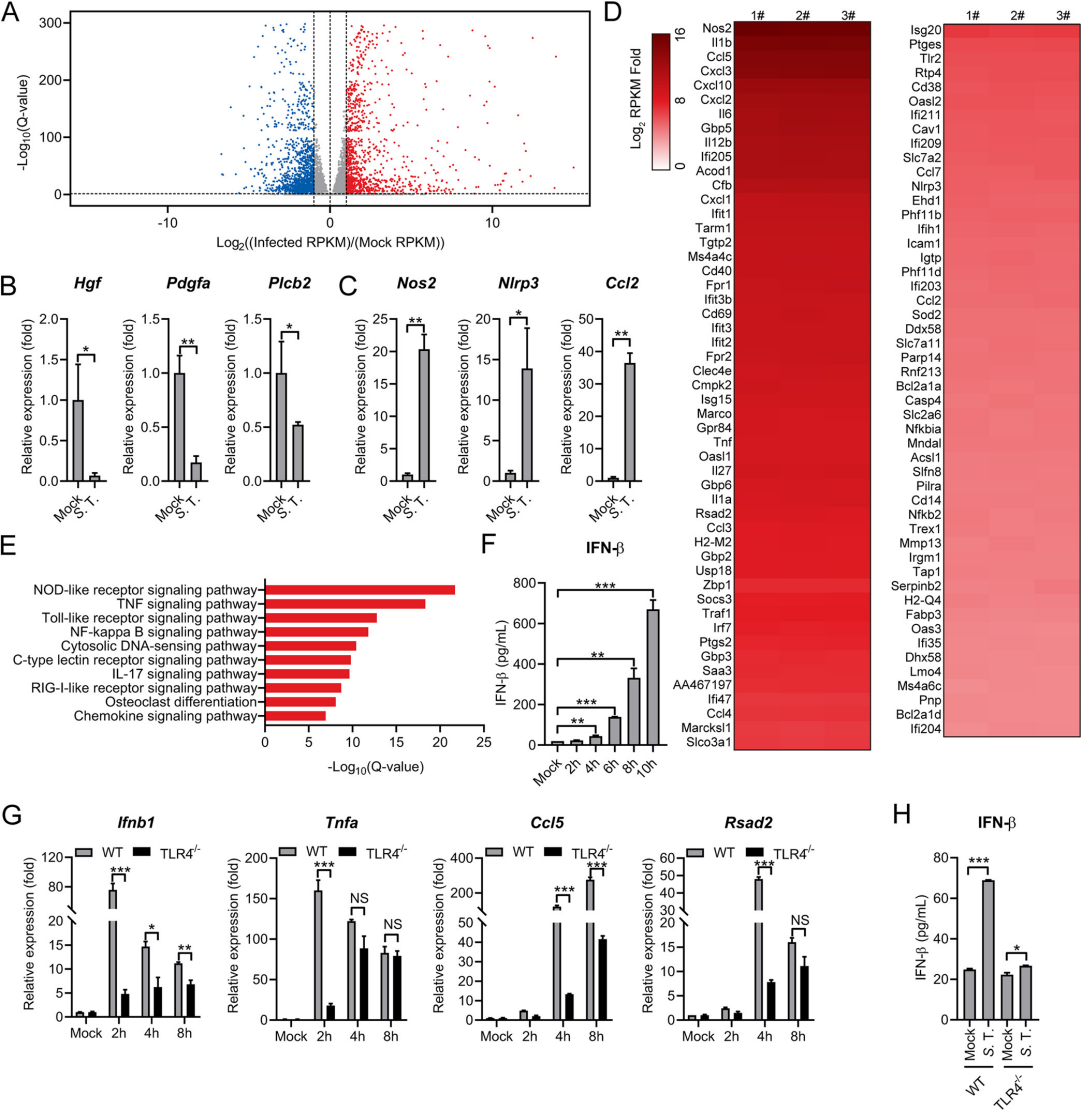
RNA-seq analysis reveals the induction of type I IFN response in *S.* Typhimurium-infected murine macrophages. (A) Volcano plot showing gene expression analysis of PMs uninfected (mock) or infected with *S.* Typhimurium for 8 h at an MOI of 10. The *x*-axis shows a fold change in gene expression by calculating log_2_(infected RPKM/mock RPKM) and the *y*-axis shows statistical significance (log_10_[Q-value]). Downregulated genes are plotted on the left (blue) and upregulated genes are on the right (red). Each treatment had 3 biological replicates. RPKM: reads per kilobase of exon model per Million mapped reads. Differentially expressed genes were those with a false discovery rate (FDR) cutoff of 0.05 and a fold change of ≥±2. (B and C) qRT-PCR analysis of gene expression of downregulated genes (B) and upregulated (C) genes in mouse PMs uninfected (mock) or infected with *S.* Typhimurium for 8 h at an MOI of 10 (*n* = 3). (D) Heatmap of RNA-seq analysis. PMs were uninfected (mock) or infected with *S.* Typhimurium for 8 h at an MOI of 10. Heatmap was made by calculating log_2_(infected RPKM/mock RPKM). Numbers 1, 2, and 3 indicate 3 biological replicates. (E) Ingenuity pathway analysis of upregulated gene expression changes in PMs infected with *S.* Typhimurium. (F) ELISA analysis of IFN-β production in mouse PMs uninfected (mock) or infected with *S.* Typhimurium for 2, 4, 6, 8, or 10 h at an MOI of 10 (*n* = 3). (G) qRT-PCR analysis of gene expression in C57BL/6 (WT) or TLR4^−/−^ mouse-derived PMs uninfected (mock) or infected with *S.* Typhimurium for 2, 4, and 8 h at an MOI of 10 (*n* = 3). (H) ELISA analysis of IFN-β production in the supernatant from C57BL/6 (WT) or TLR4^−/−^ mouse-derived PMs uninfected (mock) or infected with *S.* Typhimurium for 6 h at an MOI of 10 (*n* = 3). Data in (B) and (C) were normalized to uninfected control (mock, set as 1), Data in (G) were normalized to mock-infected C57BL/6 (WT) PMs, mock-infected TLR4^−/−^ mouse-derived PMs, respectively (mock, both set as 1). *Actin* was used as the housekeeping gene. Error bars represent ± SEM. *, *P* < 0.05; **, *P* < 0.01; ***, *P* < 0.001.

To comprehensively examine the macrophage innate immune responses to *S.* Typhimurium infection, we performed a transcriptomic analysis to assess global gene expression changes upon infection. Mouse PMs were infected with *S.* Typhimurium at an MOI of 10. At 8 h.p.i, total RNA from 3 biological replicates was collected and subjected to high-throughput RNA sequencing. Compared to uninfected control cells, over 3500 genes were found to be differentially expressed (≥2-fold) with a false discovery rate (FDR) cutoff of 0.05, with 1789 upregulated genes and 1745 downregulated genes (Fig. 2A and Table S1). To validate RNA-seq results, the expression of representative downregulated (*Hgf*, *Pdgfa*, and *Plcb2*) and upregulated (*Nos2*, *Nlrp3*, and *Ccl2*) genes was confirmed by RT-qPCR (Fig. 2B and C). In particular, a large subset of canonical proinflammatory cytokines, chemokines, inflammasome genes, and prostaglandins was potently upregulated in *S.* Typhimurium-infected macrophages (Fig. 2D). Of note, many ISGs controlled by IFNs through different IRFs (interferon regulatory factors) were also profoundly activated (Fig. 2D).

10.1128/mbio.03632-21.8 TABLE S1 RNA-seq analysis of uninfected and *S*. Typhimurium-infected macrophages. Download Table S1, XLSX file, 0.3 MB. Copyright © 2022 Xu et al. 2022 Xu et al. https://creativecommons.org/licenses/by/4.0/ This content is distributed under the terms of the Creative Commons Attribution 4.0 International license.

Further KEGG pathways enrichment analysis of DEGs (differentially expressed genes) demonstrated that the immune system and signal transduction pathways were activated in *S.* Typhimurium-infected macrophages (Fig. S1E). Unbiased canonical pathway analysis (Ingenuity pathway analysis) showed most of the upregulated genes related to innate immune signaling, including NOD-like receptor and Toll-like receptor signaling pathways (Fig. 2E). Intriguingly, the cytosolic DNA-sensing pathway and RIG-I-like receptor signaling pathway were also enriched as the most activated pathways, implying that those pathways were involved in sensing *S.* Typhimurium infection (Fig. 2E). The induction of these pathways indicates type I IFN responses were activated during infection. As expected, the IFN-β production was detected in the supernatant from *S.* Typhimurium-infected PMs by using an enzyme-linked immunosorbent assay (ELISA) (Fig. 2F). Consistently, the hallmark of type I IFN pathway activation, the phosphorylation of IRF3 and TBK1, were observed in *S.* Typhimurium infected PMs and Raw264.7 cells (Fig. S1F and G). Together, we demonstrated that *S.* Typhimurium provoked the type I IFN response in murine macrophages.

It has been reported that *S.* Typhimurium infection could stimulate type I IFN response via TLRs signaling (39). In particular, *S.* Typhimurium lipopolysaccharides (LPS) activated the production of proinflammatory cytokines via a surface innate immune receptor Toll-like receptor 4 (TLR4) (2, 3). Consistently, we showed that *S.* Typhimurium infection-induced *Ifnb1*, proinflammatory cytokines and ISG expression (Fig. 2G), and IFN-β production (Fig. 2H) were substantially attenuated in PMs from mice that lack TLR4 (TLR4^−/−^). However, it is worthy to note that the expression of these genes and production of IFN-β was not completely diminished in TLR4^−/−^ PMs in the context of *S.* Typhimurium infection. In particular, *S.* Typhimurium infection can still elicit clear *Ifnb1*, *Tnfa*, *Ccl5*, and *Rsad2* expression at 8 h.p.i (Fig. 2G). Of note, the expression of *Tnfa*, which is activated by several cytosolic innate immune sensors (40), was not affected in TLR4^−/−^ cells in response to *S.* Typhimurium infection (Fig. 2G). Moreover, a slight but statistically significant increased IFN-β production was detected in *S.* Typhimurium-infected TLR4^−/−^ PMs (Fig. 2H), suggesting TLR4-independent pathways are involved to detect *S.* Typhimurium infection. Furthermore, we used primary mouse embryonic fibroblast cells (MEFs) to detect type I IFN response during *S.* Typhimurium infection. Consistently, the lower level of type I IFN response was induced by LPS in primary MEFs compared to those in PMs (Fig. S2A) (41). With functional DNA sensing pathways (Fig. S2A), profound induction of *Ifnb1* and ISG was detected in primary MEFs during *S.* Typhimurium infection (Fig. S2B). Collectively, those results indicate that in addition to TLR4, there are TLR-independent pathways that allow the recognition of *S.* Typhimurium infection.

10.1128/mbio.03632-21.2 FIG S2 *S.* Typhimurium infection induces the type I IFN response in primary MEFs. (A) qRT-PCR analysis of gene expression in murine primary MEF cells untreated (con), treated with LPS (100 ng/mL) (LPS-MEFs), or transfected with dsDNA (1 μg/mL) (Interferon stimulatory DNA, ISD, 45 bp) (ISD-MEFs) for 6 h. PMs treated with LPS (100 ng/mL) (LPS-PMs) for 6 h were also included (*n* = 3). (B) qRT-PCR analysis of gene expression in murine primary MEF cells uninfected (mock) or infected with *S.* Typhimurium for 2, 4, 6, 8, or 10 h at an MOI of 100 (*n* = 3). Data in (A) were normalized to untreated MEFs and PMs, respectively (con, set as 1; con for PMs was not shown). Data in (B) were normalized to mock-infected control (mock, set as 1). *Actin* was used as the housekeeping gene. Error bars represent ± SEM. *, *P < *0.05; **, *P < *0.01; ***, *P < *0.001. NS, not significant. Download FIG S2, TIF file, 2.2 MB. Copyright © 2022 Xu et al. 2022 Xu et al. https://creativecommons.org/licenses/by/4.0/ This content is distributed under the terms of the Creative Commons Attribution 4.0 International license.

### *S.* Typhimurium induces type I IFN response requires TBK1-IFN axis.

**FIG 3:**
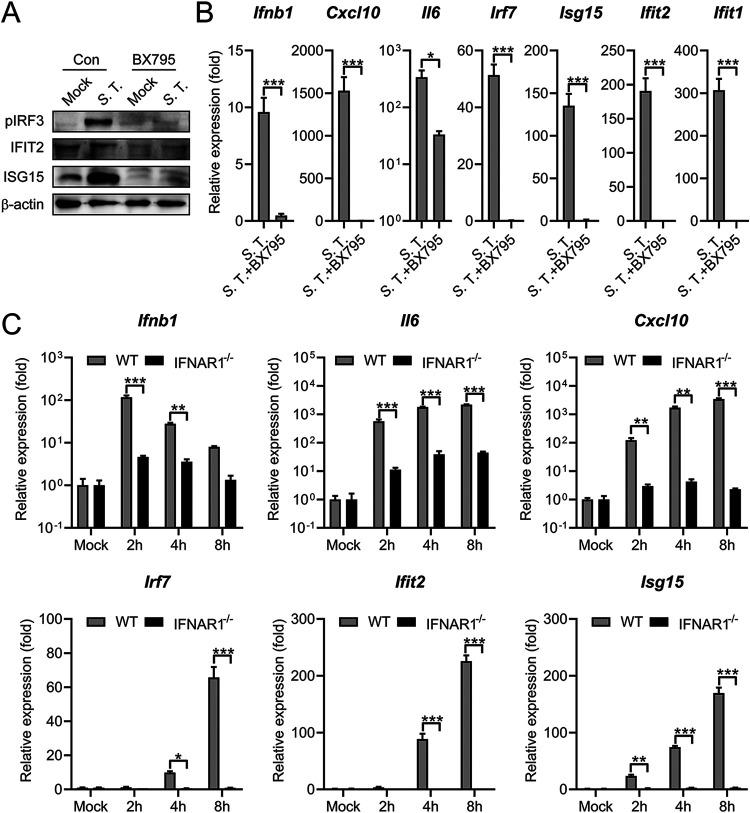
TBK1-IFN axis is required for the *S.* Typhimurium-induced type I IFN response. (A) Immunoblot analysis of protein expression in Raw264.7 cells untreated (con) or pretreated with BX795 (2 μM) for 2 h and uninfected (mock) or infected with *S.* Typhimurium for 8 h at an MOI of 100. (B) qRT-PCR analysis of gene expression in PMs untreated (con) or pretreated with BX795 (2 μM) for 2 h and uninfected (mock) or infected with *S.* Typhimurium for 8 h at an MOI of 10 (*n* = 3). (C) qRT-PCR analysis of gene expression in C57BL/6 or IFNAR1^−/−^ mouse-derived PMs uninfected (mock) or infected with *S.* Typhimurium for 2, 4, or 8 h at an MOI of 10 (*n* = 3). Data in (B) were normalized to untreated, mock-infected control and BX795-treated, mock-infected control, respectively (mock, both set as 1, not shown). Data in (C) were normalized to mock-infected C57BL/6 PMs and mock-infected IFNAR1^−/−^ PMs, respectively (mock, both set as 1). *Actin* was used as the housekeeping gene. Error bars represent ± SEM. *, *P* < 0.05; **, *P* < 0.01; ***, *P* < 0.001.

To explore other signaling pathways that mediate the *S.* Typhimurium-induced type I IFN response, a specific inhibitor of TBK1 and an inhibitor of IKKɛ, BX795 was employed (42). BX795 pretreatment leads to a substantially decreased IRF3 phosphorylation, ISG15, and IFIT2 expression in *S.* Typhimurium infected macrophages (Fig. 3A). Consistently, the gene expression of *Ifnb1*, *Il6*, *Cxcl10*, and many ISGs was also reduced in BX795-pretreated cells during infection (Fig. 3B).

The induction of type I IFNs will induce the expression of downstream ISGs in both autocrine and paracrine fashions. Autocrine sensing of type I IFN by IFN-α/β receptor (IFNAR1/2) induces ISGs expression. To probe the role of IFNAR1 in the induction of type I IFN response during *S.* Typhimurium, we examined the expression of *Ifnb1*, *Il6*, *Cxcl10*, *Irf7*, *Isg15*, and *Ifit2* in *S.* Typhimurium-infected WT or IFNAR1^−/−^ PMs by qRT-PCR at different time points postinfection. As shown in Fig. 3C, the induction of all these genes was substantially reduced in IFNAR1^−/−^ PMs at different time points postinfection. Remarkably, significantly reduced *Ifnb1* was observed in IFNAR1^−/−^ cells, suggesting an autocrine manner in *Ifnb1* induction (Fig. 3C). Together, we demonstrated that *S.* Typhimurium infection elicits a robust type I IFN response through the TBK1-IFN axis.

### *S.* Typhimurium infection provokes the cGAS-STING-dependent type I IFN response.

**FIG 4:**
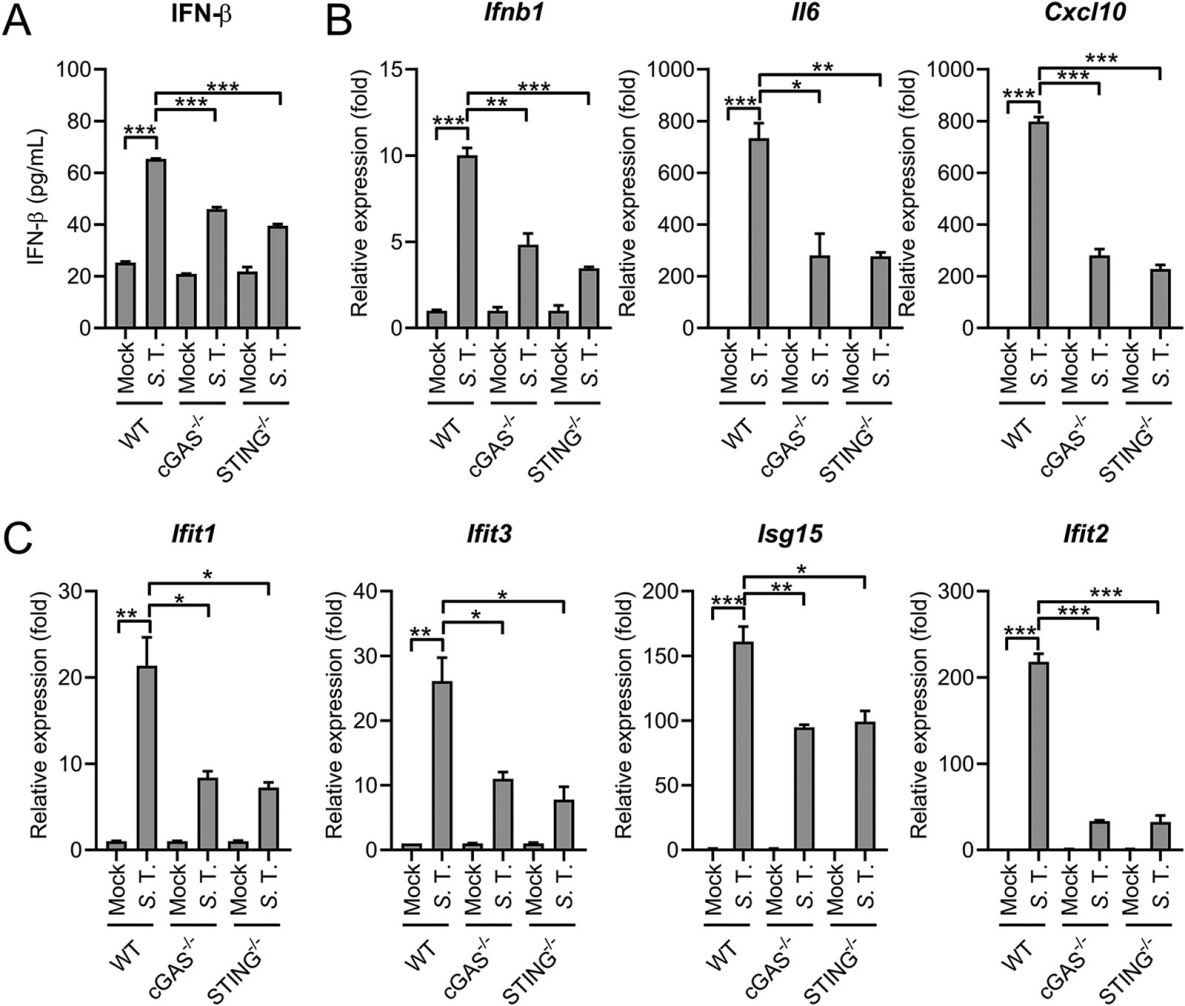
cGAS-STING contributes to *S.* Typhimurium-induced type I IFN response. (A) ELISA analysis of IFN-β production in the supernatant from C57BL/6 (WT), cGAS^−/−^ or STING^−/−^ mouse-derived PMs uninfected (mock) or infected with *S.* Typhimurium for 6 h at an MOI of 10 (*n* = 3). (B) and (C) qRT-PCR analysis of gene expression in C57BL/6 (WT), cGAS^−/−^ or STING^−/−^ mouse-derived PMs uninfected (mock) or infected with *S.* Typhimurium for 8 h at an MOI of 10 (*n* = 3). Data in (B) and (C) were normalized to mock-infected C57BL/6 (WT) PMs, mock-infected cGAS^−/−^ PMs, and mock-infected STING^−/−^ PMs, respectively (mock, all set as 1). *Actin* was used as the housekeeping gene. Error bars represent ± SEM. *, *P* < 0.05; **, *P* < 0.01; ***, *P* < 0.001.

With the observation that many cytosolic immune sensing pathways were activated during infection (Fig. 2E), we speculated cytosolic innate immune sensors may contribute to the detection of *S.* Typhimurium. Accumulating evidence suggests the cytosolic DNA-sensing pathway cGAS-STING plays an important role in inducing type I IFN response during intracellular bacterial pathogen infection (13, 19, 37, 43–46). We hypothesized that intracellular pathogenic *S.* Typhimurium could also induce the type I IFN response through this pathway. To explore this, mouse PMs derived from C57BL/6, cGAS^−/−^ or STING^−/−^ mice were infected with *S.* Typhimurium. As expected, *S.* Typhimurium-induced IFN-β production was significantly decreased in cGAS or STING deficient PMs, indicating the role of the cGAS-STING pathway in mediating *S.* Typhimurium-elicited type I IFN response (Fig. 4A and Fig. S3A). Consistently, the gene expression of *Ifnb1*, proinflammatory cytokines, and many ISGs was also significantly reduced in cGAS^−/−^ or STING^−/−^ cells (Fig. 4B), suggesting the important role of cGAS-STING in mediating *S.* Typhimurium-elicited type I IFN response.

10.1128/mbio.03632-21.3 FIG S3 cGAS-STING is required for the type I IFN response during *S.* Typhimurium infection. (A) ELISA analysis of IFN-β production in the supernatant from C57BL/6 (WT), cGAS^−/−^ or STING^−/−^ mouse-derived PMs uninfected (mock) or infected with *S*. Typhimurium for 2, 4, 6, or 8 h at an MOI of 10 (*n* = 3). (B) qRT-PCR analysis of gene expression in C57BL/6 (WT) or cGAS^−/−^ mouse-derived PMs uninfected (mock) or infected with *S*. Typhimurium for 2 and 4 h at an MOI of 10 (*n* = 3). (C) Immunoblot analysis of protein expression in C57BL/6 or STING^−/−^ mouse-derived PMs uninfected (mock) or infected with *S*. Typhimurium for 8 h at an MOI of 10, or transfected with dsDNA (Interferon stimulatory DNA, ISD) for 6 h. (D) qRT-PCR analysis of gene expression in C57BL/6 (WT) or STING^−/−^ mouse-derived PMs uninfected (mock) or infected with *S*. Typhimurium for 2 and 4 h at an MOI of 10 (*n* = 3). (E) qRT-PCR analysis of gene expression in cecum excised from *S.* Typhimurium-infected C57BL/6 (WT) or STING^−/−^ mice at 24 or 72 h postinfection (*n* = 3). Data in (B) were normalized to mock-infected C57BL/6 (WT) PMs or mock-infected cGAS^−/−^ PMs, respectively (mock, both set as 1). Data in (D) were normalized to mock-infected C57BL/6 (WT) PMs or mock-infected STING^−/−^ PMs, respectively (mock, both set as 1). Data in (E) were normalized to mock-infected C57BL/6 (WT) cecum samples and mock-infected STING^−/−^ cecum samples, respectively (mock, both set as 1). *Actin* was used as the housekeeping gene. Error bars represent ± SEM. *, *P* < 0.05; **, *P* < 0.01; ***, *P* < 0.001. Download FIG S3, TIF file, 2.0 MB. Copyright © 2022 Xu et al. 2022 Xu et al. https://creativecommons.org/licenses/by/4.0/ This content is distributed under the terms of the Creative Commons Attribution 4.0 International license.

To further profile the type I IFN response elicited by *S.* Typhimurium in cGAS^−/−^ cells, the expression pattern of many ISG early postinfection was detected. As expected, the induction of *Ifnb1* and many ISGs was significantly attenuated in cGAS^−/−^ PMs at 2 h.p.i and 4 h.p.i (Fig. S3B). Similarly, *S.* Typhimurium infection-induced expression of IRF3-responsive genes ISG15 and IFIT2 and phosphorylation of TBK1 was diminished in STING^−/−^ cells (Fig. S3C). In addition, STING^−/−^ mice derived PMs also exhibited significant lower type I IFN response during *S.* Typhimurium infection at 2 h.p.i and 4 h.p.i (Fig. S3D), suggesting the cGAS-STING pathway was also involved in the initial activation of type I IFN response during infection (Fig. S3B). Next, cecum samples were excised from PBS-treated or *S.* Typhimurium-infected WT and STING^−/−^ mice at 24 or 72 h postinfection. A marked elevated ISG induction was observed at 72 h.p.i, whereas all ISGs were induced to a lower extent in *S.* Typhimurium-infected STING^−/−^ mice (Fig. S3E). Together, these results demonstrated cGAS-STING pathway is a bona fide contributor to the host type I IFN response during *S.* Typhimurium infection.

### Activation of the STING potentiates *S.* Typhimurium-induced type I IFN response.

**FIG 5:**
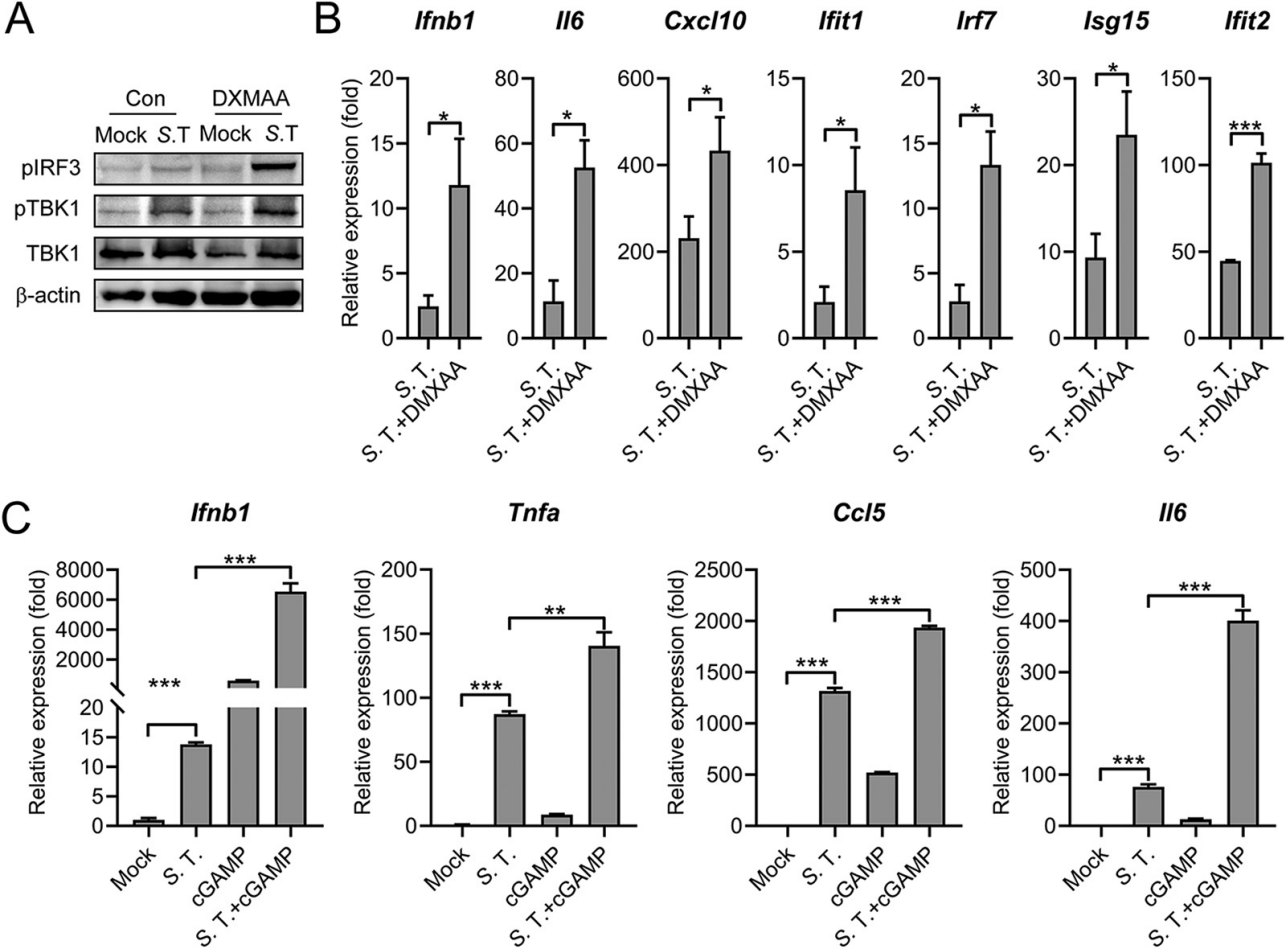
Activation of STING potentiates *S.* Typhimurium-induced type I IFN response. (A) Immunoblot analysis of protein expression in Raw264.7 untreated (con) or pretreated with DMXAA (100 μg/mL) for 12 h and uninfected (mock) or infected with *S.* Typhimurium for 8 h at an MOI of 100. (B) qRT-PCR analysis of gene expression in PMs untreated (con) or pretreated with DMXAA (100 μg/mL) for 12 h and uninfected (mock) or infected with *S*. Typhimurium for 8 h at an MOI of 10 (*n* = 3). (C) PMs were untreated or lipofectamine-transfected with 5 μM 2′3′-cGAMP (cGAMP) for 4 h and then uninfected (mock) or infected with *S.* Typhimurium at an MOI of 10. At 6 h postinfection, qRT-PCR analysis of gene expression (*n* = 3). Data in (B) were normalized to untreated, mock-infected control (mock, set as 1, not shown). Data in (C) were normalized to untreated, mock-infected control (mock, set as 1). *Actin* was used as the housekeeping gene. Error bars represent ± SEM. *, *P* < 0.05; **, *P* < 0.01; ***, *P* < 0.001.

Further experiments were performed to validate the role of STING in mediating type I IFN during *S.* Typhimurium infection. Mouse PMs were treated with 5,6-dimethylxanthenone-4-acetic acid (DMXAA, Vadimezan), a small molecule of mouse STING agonist, to activate the STING pathway (47). As shown in Fig. 5A, DMXAA treatment profoundly potentiated the *S.* Typhimurium-induced IRF3 and TBK1 phosphorylation. Furthermore, the gene expression of *Ifnb1*, proinflammatory cytokines, and ISGs was also significantly upregulated in DMXAA-treated *S.* Typhimurium-infected PMs compared with that in the untreated condition (Fig. 5A). Furthermore, cells were stimulated with 2′3′-cGAMP, the natural ligand of STING and the *S.* Typhimurium-induced expression of *Ifnb1*, proinflammatory cytokines and ISGs was also significantly activated in PMs and MEFs (Fig. 5B and Fig. S4A). Notably, the *S.* Typhimurium-induced type I IFN response was further enhanced by 2′3′-cGAMP-transfection in TLR4^−/−^ cells (Fig. S4B). Collectively, these results demonstrated that the activation of the cGAS-STING cascade potentiated the *S.* Typhimurium-induced type I IFN response.

10.1128/mbio.03632-21.4 FIG S4 Activation of the STING potentiates *S*. Typhimurium-induced type I IFN response. (A) Primary MEFs were untreated (*S*. T.) or lipofectamine-transfected with 5 μM 2′3′-cGAMP (*S*. T. + cGAMP) for 4 h and then infected with *S*. Typhimurium at an MOI of 100. At 6 h postinfection, qRT-PCR analysis of gene expression (*n* = 3). (B) TLR4^−/−^ PMs were untreated or lipofectamine-transfected with 5 μM 2′3′-cGAMP (cGAMP) for 4 h and then uninfected (mock) or infected with *S*. Typhimurium at an MOI of 10. At 6 h postinfection, qRT-PCR analysis of gene expression (*n* = 3). Data in (A) were normalized to untreated, mock-infected control and 2′3′-cGAMP-transfected, mock-infected control, respectively (mock, both set as 1, not shown). Data in (B) were normalized to untreated, mock-infected control (mock, set as 1). *Actin* was used as the housekeeping gene. Error bars represent ± SEM. *, *P* < 0.05; **, *P* < 0.01; ***, *P* < 0.001. Download FIG S4, TIF file, 0.8 MB. Copyright © 2022 Xu et al. 2022 Xu et al. https://creativecommons.org/licenses/by/4.0/ This content is distributed under the terms of the Creative Commons Attribution 4.0 International license.

### *S.* Typhimurium triggers mtDNA release to induce type I IFN response.

**FIG 6:**
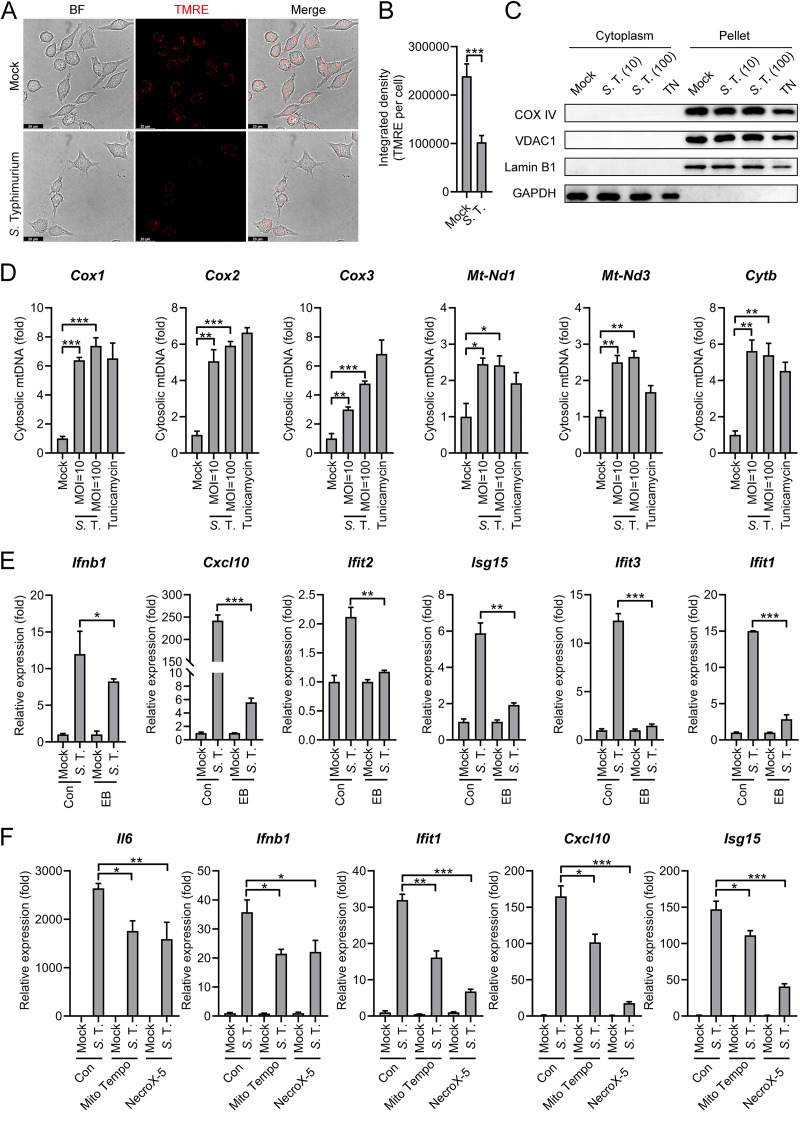
*S.* Typhimurium triggers mtDNA release to induce the type I IFN response. (A) Fluorescence microscopy analysis of mitochondrial membrane potential by assessing TMRE in Raw264.7 cells uninfected (mock) or infected with *S.* Typhimurium for 2 h at an MOI of 100. Scale bar: 20 μm. (B) The average integrated density of TMRE fluorescence. The average density was obtained from 20 individual cells for different conditions. 3 independent experiments were performed. (C) Immunoblot analysis of protein expression in different fractions in Raw264.7 cells uninfected (mock) or infected with *S.* Typhimurium for 8 h at an MOI of 10 or 100. Cells were treated with Tunicamycin (4 μg/mL) for 8 h as a positive-control for mtDNA release. (D) qPCR analysis of mtDNA in the cytosolic fraction of Raw264.7 cells uninfected (mock) or infected with *S.* Typhimurium for 8 h at an MOI of 10 or 100 (*n* = 3). (E) qRT-PCR analysis of gene expression in EB-treated and untreated (con) Raw264.7 cells uninfected (mock) or infected with *S.* Typhimurium for 8 h at an MOI of 100 (*n* = 3). (F) qRT-PCR analysis of gene expression in PMs pretreated with Mito Tempo (100 μM) or NecroX-5 (40 μM) for 2 h and uninfected (mock) or infected with *S.* Typhimurium for 6 h at an MOI of 10 (*n* = 3). Data in (D) were normalized to mock-infected control (con, set as 1). Data in (E) and (F) were normalized to untreated, mock-infected control and pretreated, mock-infected control, respectively (mock, all set as 1). *Actin* was used as the housekeeping gene. Error bars represent ± SEM. *, *P* < 0.05; **, *P* < 0.01; ***, *P* < 0.001.

Emerging studies suggest the self-DNA like mitochondrial DNA (mtDNA) abnormally present within the cytosol also stimulates the cGAS-STING pathway (17, 48). In addition, Salmonella can target mitochondria, possibly using its type III secretion system (T3SS) effectors (49–51). Therefore, we hypothesized that *S.* Typhimurium infection can lead to the release of mtDNA into cytosol that engages cGAS in macrophages. To explore this, the mitochondrial membrane potential (MMP) in *S.* Typhimurium-infected cells was measured, which acts as an indicator of mitochondrial injury. The MMP was significantly decreased during *S.* Typhimurium infection by using the TMRE (tetramethylrhodamine ethyl ester) (Fig. 6A and B), a positively charged dye that fails to retain in mitochondria has decreased membrane potential (52). Of note, although in Fig. 6A, fewer cells were captured in *S.* Typhimurium-infected condition, we calculated the average Integrated density of TMRE fluorescence from 20 different cells, respectively, with 3 independent experiments. We found that the TMRE-associated signal was significantly lower in infected cells. In addition, JC-1 dye, which forms polymers in mitochondria with normal MMP and is released from the mitochondria to form monomers with reduced MMP, was also employed (53). The formation of JC-1 monomer in *S.* Typhimurium-infected cells further confirmed the reduction of MMP (Fig. S5A).

10.1128/mbio.03632-21.5 FIG S5 *S*. Typhimurium-induced mtDNA release is involved in the induction of type I IFN. (A) Fluorescence microscopy analysis of mitochondrial membrane potential by assessing JC-1 aggregates and JC-1 monomer in HeLa uninfected (mock) or infected with *S*. Typhimurium for 2 h at an MOI of 10. Green: JC-1 monomer; Red: JC-1 aggregates. Scale bar: 75 μm. Right: fluorescence spectrophotometer analysis of the ratio of JC-1 aggregates/JC-1 monomer in HeLa cells uninfected (mock) or infected with *S*. Typhimurium for 2 h at an MOI of 10 or 100 (*n* = 3). (B) Fluorescence microscopy analysis of the localization of mitochondria and *S.* Typhimurium in Raw264.7 cells infected with *S.* Typhimurium for 2 h at an MOI of 100. Hoechst33342: nuclei; GFP, Mito-tracker green to indicate mitochondria; mCherry, *S.* Typhimurium carries a mCherry expressing plasmid. Scale Bar: 20 μm. (C) qPCR analysis of mtDNA in the cytosolic fraction of C57BL/6 (WT) or TLR4^−/−^ mouse-derived PMs uninfected (mock) or infected with *S*. Typhimurium for 8 h at an MOI of 100 (*n* = 3). (D) qPCR analysis of the total mtDNA in EB-treated and untreated (con) Raw264.7 cells (*n* = 3). (E) qPCR analysis of mtDNA in the cytosolic fraction of PMs pretreated with Mito Tempo (100 μM) or NecroX-5 (40 μM) for 2 h and uninfected (mock) or infected with *S*. Typhimurium for 6 h at an MOI of 100 (*n* = 3). (F) qRT-PCR analysis of gene expression in TLR4^−/−^ PMs pretreated with Mito Tempo (100 μM) for 2 h and uninfected (mock) or infected with *S*. Typhimurium for 6 h at an MOI of 10 (*n* = 3). Data in (C) were normalized to uninfected C57BL/6 (WT) and TLR4^−/−^ PMs, respectively (mock, both set as 1). Data in (D) were normalized to untreated control (con, set as 1). Data in (E) were normalized to untreated mock-infected control (mock, set as 1). Data in (F) were normalized to untreated, mock-infected control and Mito Tempo pretreated, mock-infected control, respectively (mock, both set as 1). *Actin* was used as the housekeeping gene. Error bars represent ± SEM. *P < 0.05; **P < 0.01; ***P < 0.001. NS, not significant. Download FIG S5, TIF file, 2.4 MB. Copyright © 2022 Xu et al. 2022 Xu et al. https://creativecommons.org/licenses/by/4.0/ This content is distributed under the terms of the Creative Commons Attribution 4.0 International license.

Next, to directly probe the release of mtDNA into the cytosol during *S.* Typhimurium infection. Different fractions of *S.* Typhimurium infected cells were separated and the immunoblot analysis confirmed the isolation of the cytoplasm fraction, with no cross-contamination of mitochondria-associated proteins in the cytosolic fractions (Fig. 6C). *S.* Typhimurium infection results in the release of mtDNA into the cytosol, as manifested by a higher level of mitochondrial genes in cytosolic fractions (Fig. 6D), although no colocalization between *S.* Typhimurium and mitochondria was detected (Fig. S5B). The Tunicamycin that inhibits GlcNAc phosphotransferase is used as a positive-control for mtDNA release (54). Interestingly, the release of mtDNA into the cytosol was also detected in TLR4^−/−^ PMs (Fig. S5C).

To explore the requirement of mtDNA for *S.* Typhimurium-induced type I IFN response, Raw264.7 cells were treated with Ethidium bromide (EtBr) to delete the mtDNA (54). MtDNA depletion in EtBr-treated Raw264.7 cells was confirmed by qPCR (Fig. S5D). After removing the EtBr, both untreated and EtBr-treated cells were infected by *S.* Typhimurium. The induction of type I IFN response was significantly attenuated in EtBr-treated cells (Fig. 6E). This result proved that mtDNA is important for the activation of type I IFN response during *S.* Typhimurium in murine macrophages. To further explore the release of mtDNA could active type I IFN response, Raw264.7 cells were treated with a mitochondrial-targeted antioxidant, Mito Tempo ([2-(2,2,6,6-tetramethylpiperidin-1-oxyl-4-ylamino)-2-oxoethyl] triphenylphosphonium chloride), to reduce the release of mtDNA into the cytoplasm (Fig. S5E) (55). Notably, *S.* Typhimurium-induced type I IFN response was significantly decreased in Mito Tempo treated WT cells, further supporting the notion that mtDNA release elicited the type I IFN response (Fig. 6F). Importantly, this Mito Tempo also inhibits *S.* Typhimurium-induced type I IFN response in TLR4^−/−^ PMs (Fig. S5F). Furthermore, NecroX-5, an inhibitor that scavenges mitochondrial reactive oxygen (56), also reduced mtDNA release and type I IFN response during *S.* Typhimurium infection (Fig. 6F and Fig. S5E). Collectively, these results indicated that *S.* Typhimurium infection induces the release of mtDNA to the cytosol and mtDNA can act as the danger signal to induce type I IFN pathways.

### Bacterial DNA binds to cGAS during *S.* Typhimurium infection.

**FIG 7:**
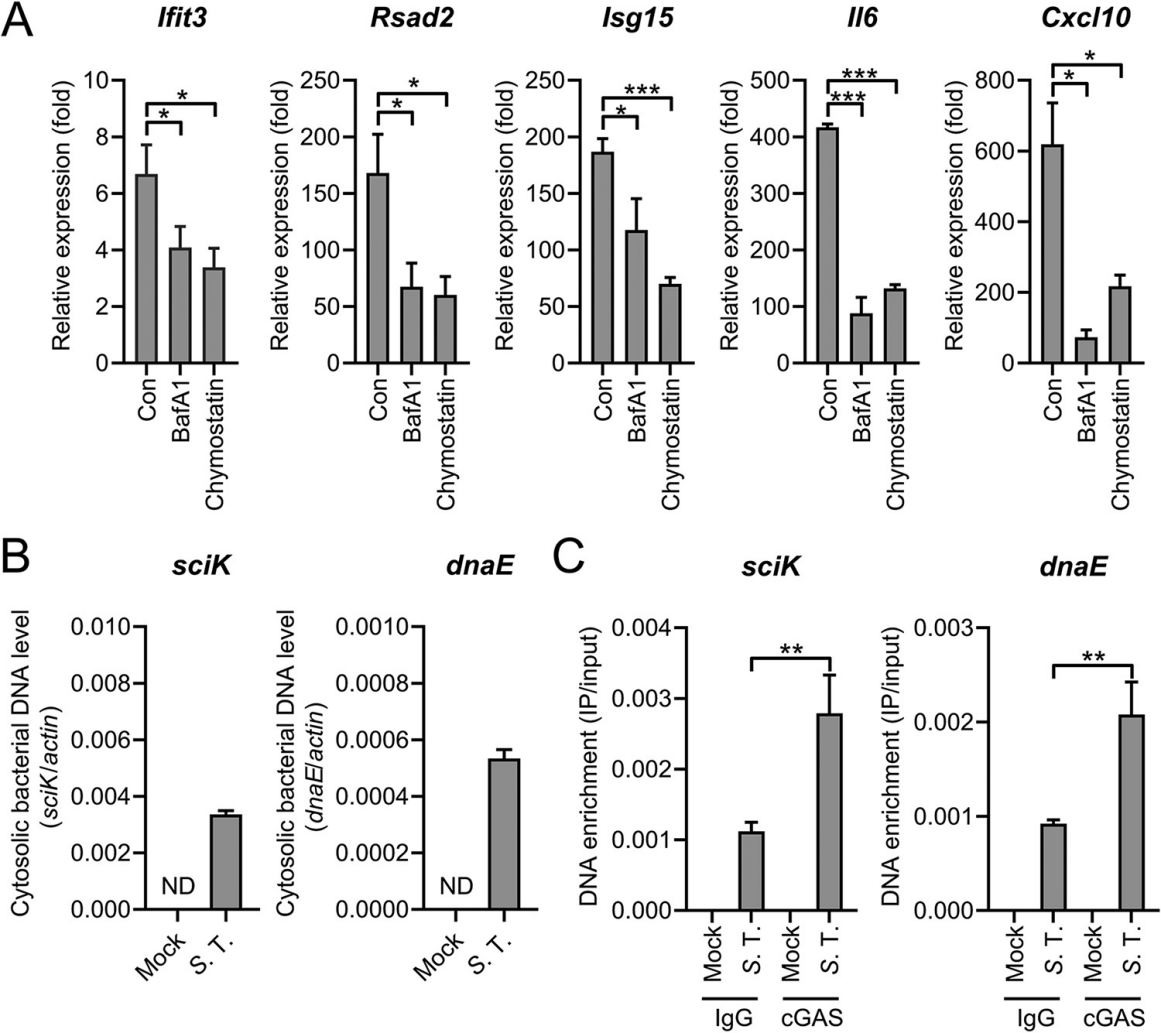
Bacterial DNA binds to cGAS during *S.* Typhimurium infection. (A) qRT-PCR analysis of gene expression in PMs untreated (con) or pretreated with BafA1 (200 nM) or chymostatin (100 μM) for 2 h and uninfected (mock) or infected with *S.* Typhimurium for 8 h at an MOI of 10 (*n* = 3). (B) qPCR analysis of *S.* Typhimurium-derived sequences (*dnaE* and *sciK*) in the cytosolic fraction of PMs uninfected (mock) or infected with *S.* Typhimurium for 8 h at an MOI of 100 (*n* = 3). ND, not detected. (C) Raw264.7 cells were uninfected or infected with *S.* Typhimurium for 8 h at an MOI of 100, then immunoprecipitated with IgG or cGAS antibody. qPCR of *S.* Typhimurium-derived sequences (*sciK* and *dnaE*) from DNA isolated from IPs (*n* = 3). Data in (A) were normalized to untreated, mock-infected control and BafA1-treated, mock-infected, and chymostatin-treated, mock-infected, respectively (mock, all set as 1, not shown). Quantities in (C) were normalized to inputs. *Actin* was used as the housekeeping gene. Error bars represent ± SEM. *, *P* < 0.05; **, *P* < 0.01; ***, *P* < 0.001.

Pathogenic cytoplasmic DNAs bind to cGAS to produce 2′3′-cGAMP that activates STING and downstream signaling pathways (48, 57). Direct transfection of *S.* Typhimurium genomic DNA induces type I IFN response in a STING-dependent fashion in macrophages (Fig. S6A), suggesting the bacterial genomic DNA may act as a PAMPs. Moreover, we pretreated PMs with bafilomycin A1 (BafA1, a specific inhibitor of the vacuolar H^+^ ATPase that interferes with endosomal acidification and changes the formation of phagolysosomes) and chymostatin (an inhibitor of lysosomal serine and cysteine proteinases) (58) and found that those pretreated cells exhibited reduced type I IFN induction during *S.* Typhimurium infection (Fig. 7A). This indicates the degradation of bacteria in phagolysosomes contributes to the activation of type I IFN response during infection.

10.1128/mbio.03632-21.6 FIG S6 *S*. Typhimurium DNA induces type I IFN response and the cGAS-STING pathway constrains *S*. Typhimurium infection. (A) qRT-PCR analysis of gene expression in C57BL/6 (WT) or STING^−/−^ mouse-derived PMs transfected with *S*. Typhimurium genomic DNA (1 μg per well in 24-well plate) for 6 h (*n* = 3). (B) Immunoblotting of lysates of Raw264.7 cells was uninfected or infected with S. Typhimurium for 8 h at an MOI of 100, then immunoprecipitated with IgG or cGAS antibody. (C) qPCR of mtDNA sequences (*Cox1, Cox2*, and *Cox3*) from DNA isolated from IPs in (B) (*n* = 3). (D) Fluorescence microscopy analysis of C57BL/6 or cGAS^−/−^ mouse-derived PMs infected with *S*. Typhimurium for 2 h at an MOI of 10. Hoechst33342: nuclei, GFP, *S*. Typhimurium carries a GFP expressing plasmid. Scale bar: 75 μm. The ratio of GFP fluorescence integrated density to Hoechst33342 fluorescence integrated density is on the right (*n* = 3). (E) qPCR analysis of *S*. Typhimurium 16S *rRNA* level in primary MEFs untreated (con) or pretreated with DMXAA (100 μg/mL) for 12 h, or pretreated lipofectamine-transfected with 5 μM 2′3′-cGAMP (cGAMP) for 4 h and then infected with *S*. Typhimurium for 8 h at an MOI of 100 (*n* = 3). (F) qPCR analysis of *S*. Typhimurium 16S *rRNA* level in TLR4^−/−^ PMs untreated (con) or pretreated lipofectamine-transfected with 5 μM 2′3′-cGAMP (cGAMP) for 4 h and infected with *S*. Typhimurium for 8 h at an MOI of 100 (*n* = 3). Data in (A) were normalized to mock-transfected C57BL/6 (WT) PMs and mock-transfected STING^−/−^ PMs, respectively (mock, both set as 1). Quantities in (C) were normalized to inputs. Data in (E and F) were normalized to untreated *S*. Typhimurium-infected control (con, set as 1). Error bars represent ± SEM. *, *P* < 0.05; **, *P* < 0.01; ***, *P* < 0.001. Download FIG S6, TIF file, 1.5 MB. Copyright © 2022 Xu et al. 2022 Xu et al. https://creativecommons.org/licenses/by/4.0/ This content is distributed under the terms of the Creative Commons Attribution 4.0 International license.

Next, we probed the *S.* Typhimurium genes in the cytosolic fraction during *S.* Typhimurium infection. As shown in Fig. 7B, *S.* Typhimurium DNA was detected in the cytosolic fraction during *S.* Typhimurium infection. Together with the detection of mtDNA in the cytosolic fraction (Fig. 6D), these results suggest both mtDNA and *S.* Typhimurium DNA contribute to cGAS activation. To further explore factors that bind to cGAS during *S.* Typhimurium infection, a coimmunoprecipitation assay that can detect the DNA sequences enriched in cGAS was employed (19, 31). Successful immunoprecipitation of cGAS was confirmed by immunoblotting (Fig. S6B). Notably, two *S.* Typhimurium genes *sciK* and *dnaE* were detected in cGAS compared to IgG controls (Fig. 7C), suggesting *S.* Typhimurium DNA also binds to cGAS during infection. Subsequently, to distinguish the contribution of mtDNA and bacterial genomic DNA in activating cGAS, we aimed to measure mtDNA in cGAS immunoprecipitates. However, all cGAS immunoprecipitates were positive for mtDNA sequences and the abundance is similar (Fig. S6C). This may be because the strong lysis buffer causes the deconstruction of mitochondria. Together, physical interactions between cGAS and *S.* Typhimurium DNA have been identified in the context of infection. However, the relative contribution of bacterial DNA and mtDNA to cGAS activation needs further investigation.

### cGAS-STING pathway contributes to host defenses against *S.* Typhimurium infection.

**FIG 8:**
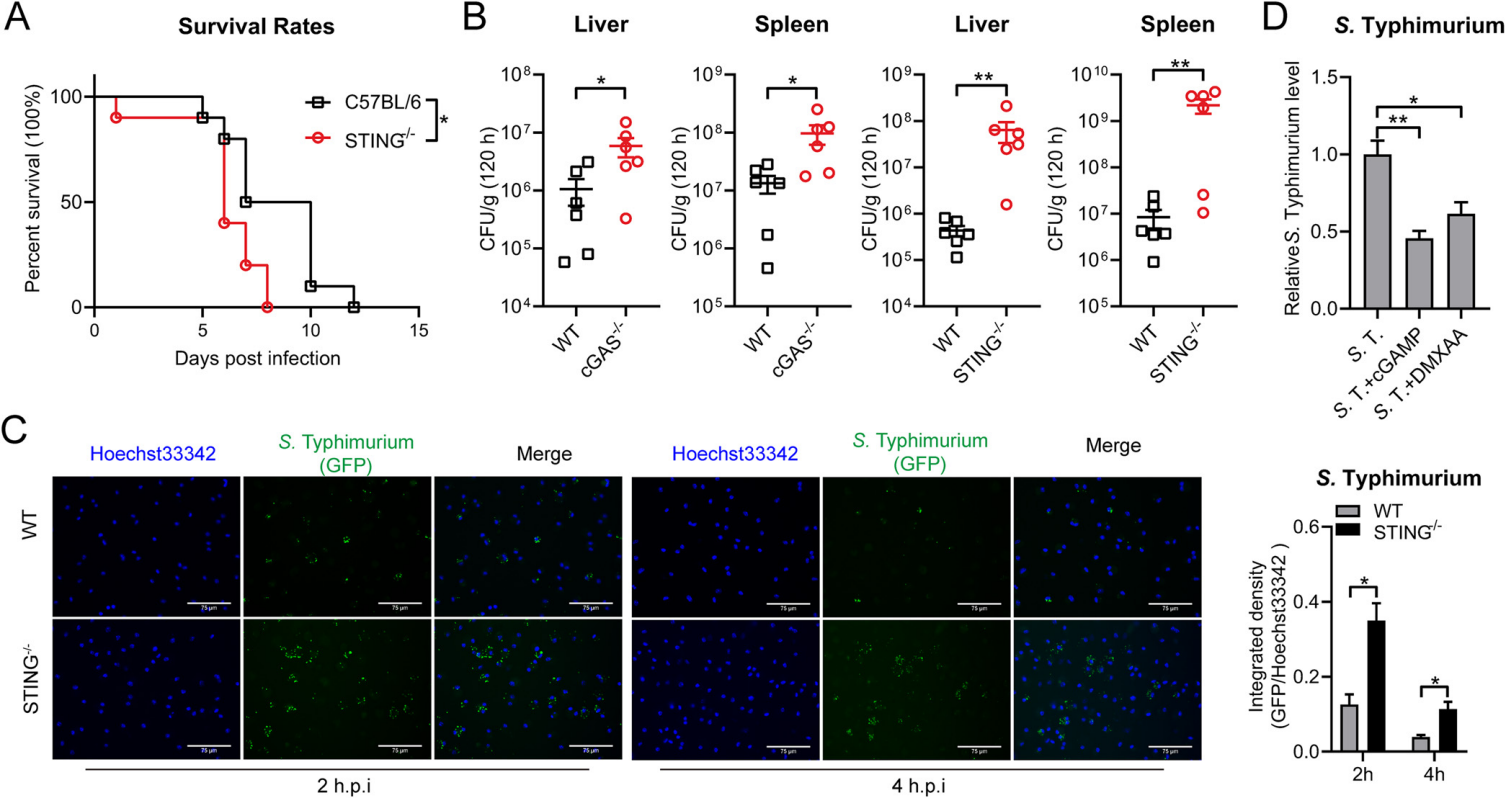
cGAS-STING contributes to host defense against *S.* Typhimurium infection. (A) and (B) C57BL/6, cGAS^−/−^, STING^−/−^ mice were intragastrically inoculated with 1 × 10^8^ CFU *S.* Typhimurium. (A) The survival rate of the mice was determined (C57BL/6, *n* = 10; STING^−/−^, *n* = 10). (B) Homogenates of the liver and spleen were plated to determine the bacterial CFU counts per gram in the indicated organs at 120 h postinfection (*n* = 6). (C) Fluorescence microscopy analysis of C57BL/6 or STING^−/−^ mouse-derived PMs infected with *S.* Typhimurium for 2 h or 4 h at an MOI of 10. Hoechst33342: nuclei, GFP, *S.* Typhimurium carry a GFP expressing plasmid. Scale bar: 75 μm. The ratio of GFP fluorescence integrated density to Hoechst33342 fluorescence integrated density is on the right (*n* = 3). (D) qRT-PCR analysis of *S.* Typhimurium *16S* rRNA level in PMs untreated (con) or pretreated with DMXAA (100 μg/mL) for 12 h, or pretreated lipofectamine-transfected with 5 μM 2′3′-cGAMP (cGAMP) for 4 h and infected with *S.* Typhimurium for 8 h at an MOI of 100 (*n* = 3). Data in (D) were normalized to untreated *S.* Typhimurium-infected control (*S*. T, set as 1). *Actin* was used as the housekeeping gene. Error bars represent ± SEM. *, *P* < 0.05; **, *P* < 0.01.

We further explored whether cGAS-STING contributes to the host defense against *S.* Typhimurium infection. To this end, WT and STING^−/−^ mice were intragastrically infected with 1 × 10^8^ CFU (CFU) of *S.* Typhimurium and the lethality was monitored over time. As shown in Fig. 8A, STING^−/−^ mice showed an early mortality rate compared with WT mice. On day 6, more than 60% of STING^−/−^ mice had died while the rate in WT mice was less than 20%, which is consistent with the previous study (59). Furthermore, we examined the bacterial load in different tissues of WT, cGAS^−/−^ and STING^−/−^ mice at 120 h postinfection. Significantly increased bacterial burden was detected from the spleen and liver of cGAS^−/−^ and STING^−/−^ mice, further fostering the role of cGAS-STING in defending *S.* Typhimurium infection (Fig. 8B).

To probe the role of cGAS-STING in effecting intracellular growth of *S.* Typhimurium in murine macrophages, *S.* Typhimurium with a GFP plasmid was used to infect WT, cGAS^−/−^ or STING^−/−^ PMs and the GFP signal in *S.* Typhimurium-infected cells was imaged. Fluorescence microscopy illustrated that the *S.* Typhimurium-infected cGAS^−/−^ and STING^−/−^ cells contained stronger GFP fluorescence compared with those *S.* Typhimurium-infected WT cells (Fig. 8C and Fig. S6D). These findings demonstrated that the lack of cGAS-STING led to an improved *S.* Typhimurium infection in mice models and intracellular growth in murine macrophages.

To explore whether the activation of the cGAS-STING pathway could eradicate intracellular *S.* Typhimurium growth, STING agonist DMXAA was used to pretreat cells and the reduced *S.* Typhimurium *16s* RNA level was obtained in DMXAA-treated PMs and MEFs (Fig. 8D). Furthermore, the 2′3′-cGAMP transfection induced STING activation also decreased intracellular *S.* Typhimurium growth level in PMs (Fig. 8D) and primary MEFs (Fig. S6E). Intriguingly, cGAMP transfection in TLR4^−/−^ PMs also decreased intracellular *S.* Typhimurium growth (Fig. S6F). Taken together, these results proved that cGAS-STING plays an important role in the host defense against *S.* Typhimurium infection.

## DISCUSSION

Salmonella induces a robust innate immune response in a TLR-dependent manner (3, 46). In addition to TLRs, cytosolic NLRs, including NLRP3 and NLRC4 are both activated during *S.* Typhimurium infection (5) and *S.* Typhimurium mRNA might act as a danger signal that induces the activation of NLRP3 (10). We also noticed macrophages derived from mice that lack the surface receptors TLR4 exhibited substantially attenuated type I IFN responses during *S.* Typhimurium infection (Fig. 2). However, whether other innate immune sensing pathways are involved during *S.* Typhimurium in macrophages remains elusive. In this study, we showed that the cytosolic surveillance pathway cGAS-STING was critically involved in the induction of the type I IFN response through the TBK1-IFN cascade.

To delineate the contribution of the different signaling pathways that mediate the type I IFN response during infection, the cGAS^−/−^, STING^−/−^ and TLR4^−/−^ mice derived PMs were used to infect *S.* Typhimurium and the type I IFN and ISG induction was measured at different time postinfection. The lack of TLR4 substantially attenuated the type I IFN and ISG expression from 2 h.p.i to 8 h.p.i (Fig. S2C). However, there is still significant induction of *Ifnb1*, proinflammatory cytokines, and ISGs in TLR4^−/−^ cells, suggesting the TLR-independent recognition mechanisms during *S.* Typhimurium infection. The release of mtDNA in TLR4^−/−^ PMs (Fig. S5F) and reduced type I IFN response in Mito Tempo treated TLR4^−/−^ PMs (Fig. S6B) further underscored the TLR-independent mechanism. Intriguingly, compared to WT cells, the type I IFN response was also significantly reduced in cGAS^−/−^ or STING^−/−^ cells, which is consistent with a previous study that demonstrated a clear reduction of both IFN-β and IFIT1 mRNAs in *S.* Typhimurium-infected cGAS^−/−^ and STING^−/−^ BMDMs (19). In particular, the *S.* Typhimurium-induced *Ifnb1* expression was attenuated in cGAS^−/−^ or STING^−/−^ PMs from 2 h.p.i, suggesting the cGAS-STING pathway was also involved in the initial activation of type I IFN response during infection.

It is not surprising that intracellular bacteria induce host innate immune response in STING-dependent manners since bacterial CDNs like c-di-GMP or c-di-AMP are efficiently recognized by STING, which leads to the production of type I IFN (12–14, 60). However, the induction of type I IFN by most bacteria is largely dependent on cGAS (19, 22–31). This may be due to the possibility that bacterial CDNs are constrained in bacterial cells, which are not easily accessed to the cytoplasm. In addition, c-di-GMP in intracellular *S.* Typhimurium was maintained at low levels (61), possibly to avoid the cytosolic detection of bacterial infection.

Both pathogenic DNAs and self-DNA like mitochondrial DNA (mtDNA) within the cytosol can stimulate the cGAS-STING pathway (17, 48, 57). The *S.* Typhimurium genomic DNA may also activate cGAS, supported by the observation that the transfection of *S.* Typhimurium DNA into macrophages can also provoke type I IFN response (Fig. S6A) and bafilomycin A1 and chymostatin pretreated reduced type I IFN induction during infection (Fig. 7A). Speculatively, phagocytosis of *S.* Typhimurium may liberate some genomic DNA as PAMPs from bacteria. Coupled with this speculation, we also detected *S*. Typhimurium genes in the cytosolic fraction and showed physical interactions between cGAS and *S.* Typhimurium DNA in the context of infection (Fig. 7B and C). This observation indicates *S.* Typhimurium DNA also contributes to cGAS activation.

Concomitantly, we demonstrated that mtDNA is also an important stimulus that activates the cGAS-STING pathway during *S.* Typhimurium infection (Fig. 6 and Fig. S5). Importantly, in EtBr-treated mtDNA depletion cells, induction of type I IFN response was significantly attenuated during *S.* Typhimurium infection (Fig. 6E), further fostering the notion that mtDNA is important for the activation of type I IFN response. However, the temptation to measure mtDNA in the cGAS immunoprecipitates to determine the abundance of mtDNA enriched in cGAS was not succeeded, making it difficult to distinguish the relative contribution of mtDNA and *S.* Typhimurium DNA in cGAS activation, and such a question needs further investigations.

Intriguingly, the release of mtDNA into cytosol can be induced by internalized bacterial endotoxin LPS, which activated the Gasdermin D to form pores in mitochondria (62). Here, we demonstrated *S.* Typhimurium infection results in the release of mtDNA into the cytosol (Fig. 6D) as internalized *S.* Typhimurium might induce mtDNA release through Gasdermin D. In addition, it has been reported that Salmonella can target mitochondria by deploying T3SS effectors (49). *S.* Typhimurium secreted a T3SS effector SipB with membrane fusion activity to target mitochondria (50). Another T3SS effector SopA was also proved to target mitochondria in HeLa or COS cells (51). These pieces of evidence imply that *S.* Typhimurium may cause mitochondria damage that led to the release of mtDNA. Similarly, Mycobacterium abscessus and Streptococcus pneumoniae infection also lead to the release of mtDNA into the cytosol of murine macrophages, which activates the cGAS-STING-dependent type I IFN production (55, 63). Building on this information, it is likely that the liberating of mtDNA into the cytosol may represent a universal mechanism to activate the cGAS-STING pathway during intracellular bacterial infection, albeit further experimental evidence was required to confirm this notion.

It has been shown that STING^−/−^ mice are highly susceptible to *S.* Typhimurium infection compared with WT animals with the perspective of the protective role of STING in regulating intestinal homeostasis (59). In addition, the STING-IRF1 signaling axis is required for the control of mucosal T_H_17 cell responses that have protective effects against *S.* Typhimurium infection (64). In particular, the uptake and transport of *S.* Typhimurium to MLNs (mesenteric lymph nodes) were slightly enhanced in STING^−/−^ mice compared to WT mice, albite no statistical significance (64). In this study, *in vivo*, and *in vitro* experiments showed the cGAS-STING pathway was potently implicated in the host defense against *S.* Typhimurium infection. We further demonstrated that the activation of the STING potently inhibits *S.* Typhimurium growth (Fig. 8D and Fig. S6E and F). Furthermore, we also noticed that in TLR4^−/−^ PMs, *S.* Typhimurium-induced type I IFN response was further enhanced by 2′3′-cGAMP-transfection and cGAMP transfection also inhibited *S.* Typhimurium infection (Fig. S4B and S6F), suggesting the cGAS-STING pathway plays an important role in the host defense against *S.* Typhimurium infection in addition to TLR4 pathway. Type I IFN production is the main feature of cGAS-STING activation, but both protective and detrimental role of type I IFN has been found during *S.* Typhimurium infection (65, 66). Thus, the underlying mechanisms of cGAS-STING against *S.* Typhimurium infection need further investigations.

**FIG 9:**
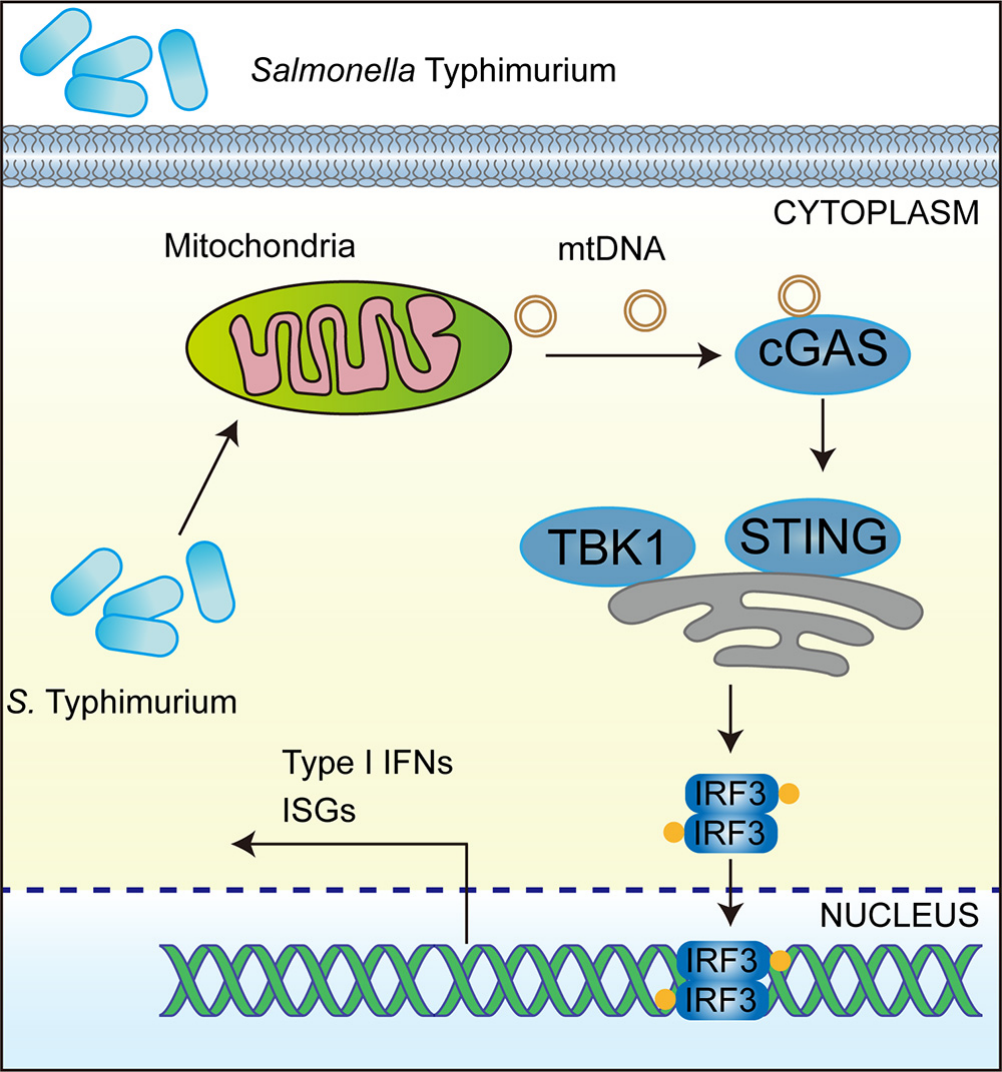
*S.* Typhimurium triggers the cGAS-STING-dependent type I IFN response by inducing mtDNA release. In murine macrophages, *S.* Typhimurium infection led to the release of mtDNA into cytosol that possibly engages cytosolic DNA sensor cGAS. The activated cGAS led to the activation of immune adaptor protein STING and phosphorylation of kinase TBK1, which activates the transcription factor IRF3. Translocation of IRF3 resulted in transcriptional induction of the type I IFN response.

In conclusion, we demonstrated that in addition to TLR4-dependent response, *S.* Typhimurium could induce type I IFN response in a STING-dependent manner by triggering mtDNA release (Fig. 9). This study highlighted the important role of cGAS-STING in meditating type I IFN response and host defense in the context of *S.* Typhimurium infection. Furthermore, this study uncovered a mechanism by which type I IFN response is elicited through the liberating of mtDNA into the cytosol during *S.* Typhimurium infection. Our new paradigms deciphering the requirement of the cGAS-STING pathway largely expanded the current understanding of *S.* Typhimurium pathogenesis and innate immunity.

## MATERIALS AND METHODS

### Mice.

Mice used in this study were on a C57BL/6 background. WT C57BL/6 mice were purchased from the Animal Center of Xi’an Jiaotong University. cGAS^−/−^, STING^−/−^ mice, and IFNAR1^−/−^ mice based on the C57BL/6 background were kindly provided by Zhengfan Jiang (67). C57BL/10ScNJGpt (TLR4^−/−^) and wild-type C57BL/10JGpt mice were purchased from GemPharmatech, Jiangsu, China. All the mouse experimental procedures were performed under the Regulations for the Administration of Affairs Concerning Experimental Animals approved by the State Council of the People’s Republic of China. The protocol was approved by the Animal Welfare and Research Ethics Committee of Northwest A&F University (protocol number: NWAFUSM2018001). Male and female mice were sex-matched and used at 6 to 12 weeks of age and were kept under specific pathogen-free conditions.

### *S.* Typhimurium infection.

*S.* Typhimurium was cultured in LB overnight at 37°C with 100 μg/mL streptomycin or 50 μg/mL kanamycin (for strains carried pKT100 plasmids). The following day, pelleted bacteria at 4500 rpm by centrifugation and washed bacteria 3 times with sterile PBS and resuspended in RPMI 1640 or DMEM medium. When the OD_600_ = 2.5, there are approximately 10^8^ CFU *S.* Typhimurium in 1 mL resuspension. Different volumes of bacterial resuspension were mixed with antibiotic-free RPMI 1640 or DMEM to obtain the desired multiplicity of infection (MOI). Peritoneal macrophages (PMs) were washed 3 times with sterile PBS before infection and cultured in RPMI 1640 medium without FBS and penicillin-streptomycin before infection. The cells were infected at indicated MOI and then the plate was centrifuged at 300 × *g* for 5 min to synchronize infection (34). Infected cells were incubated at 37°C for 20 min and then the infection medium was removed, and the cells were washed 3 times by sterile PBS and replaced with RPMI 1640 containing FBS (10%) and gentamicin (200 μg/mL) for indicated times to kill extracellular bacteria. Raw264.7, HeLa, and primary mouse embryonic fibroblast (MEF) cells were cultured in DMEM and changed to antibiotic-free and FBS-free DMEM medium. Infected cells were incubated at 37°C for 2 h and then the infection medium was removed and replaced with DMEM containing FBS (10%) and gentamicin (200 μg/mL) for indicated times to kill extracellular bacteria.

For mice infection, food and water were deprived 12 h before infection. Mid-exponential-phase *S.* Typhimurium was collected and washed twice in sterile PBS. Each mouse was intragastrically infected with 1 × 10^8^ CFU *S.* Typhimurium by ball-tipped feeding needles and food and water were resupplied from 12 h postinfection. For survival assays, the daily survival was observed and recorded for 2 weeks to calculate the death rate of the mice in different groups (59). For the detection of bacterial load in different organs, mice were sacrificed by carbon dioxide asphyxiation followed by cervical dislocation at 120 h postinfection. Different tissues were weighed and homogenized in 0.9% NaCl, and serial dilutions of the homogenates were plated on LB plates with 200 μg/mL streptomycin.

The rest of the Materials and Methods are described in detail in Text S1 and Table S2. The uncropped versions of immunoblotting results were provided in Fig. S7.

10.1128/mbio.03632-21.7 FIG S7 Unprocessed western blots results. Download FIG S7, JPG file, 1.07 MB. Copyright © 2022 Xu et al. 2022 Xu et al. https://creativecommons.org/licenses/by/4.0/ This content is distributed under the terms of the Creative Commons Attribution 4.0 International license.

10.1128/mbio.03632-21.9 TABLE S2 Bacterial strains, cells, mice, plasmids, primers, reagents, and resources used in this study. Download Table S2, XLSX file, 0.02 MB. Copyright © 2022 Xu et al. 2022 Xu et al. https://creativecommons.org/licenses/by/4.0/ This content is distributed under the terms of the Creative Commons Attribution 4.0 International license.

10.1128/mbio.03632-21.10 TEXT S1 Supplemental materials and methods. Download Text S1, DOCX file, 0.05 MB. Copyright © 2022 Xu et al. 2022 Xu et al. https://creativecommons.org/licenses/by/4.0/ This content is distributed under the terms of the Creative Commons Attribution 4.0 International license.
