# Supplementary figures and images for: Salmonella Induces the cGAS-STING-Dependent Type I Interferon Response in Murine Macrophages by Triggering mtDNA Release
Source: mBio. 2022 May 23;13(3):e03632-21. doi: 10.1128/mbio.03632-21 (PMC9239183; doi:10.1128/mbio.03632-21)

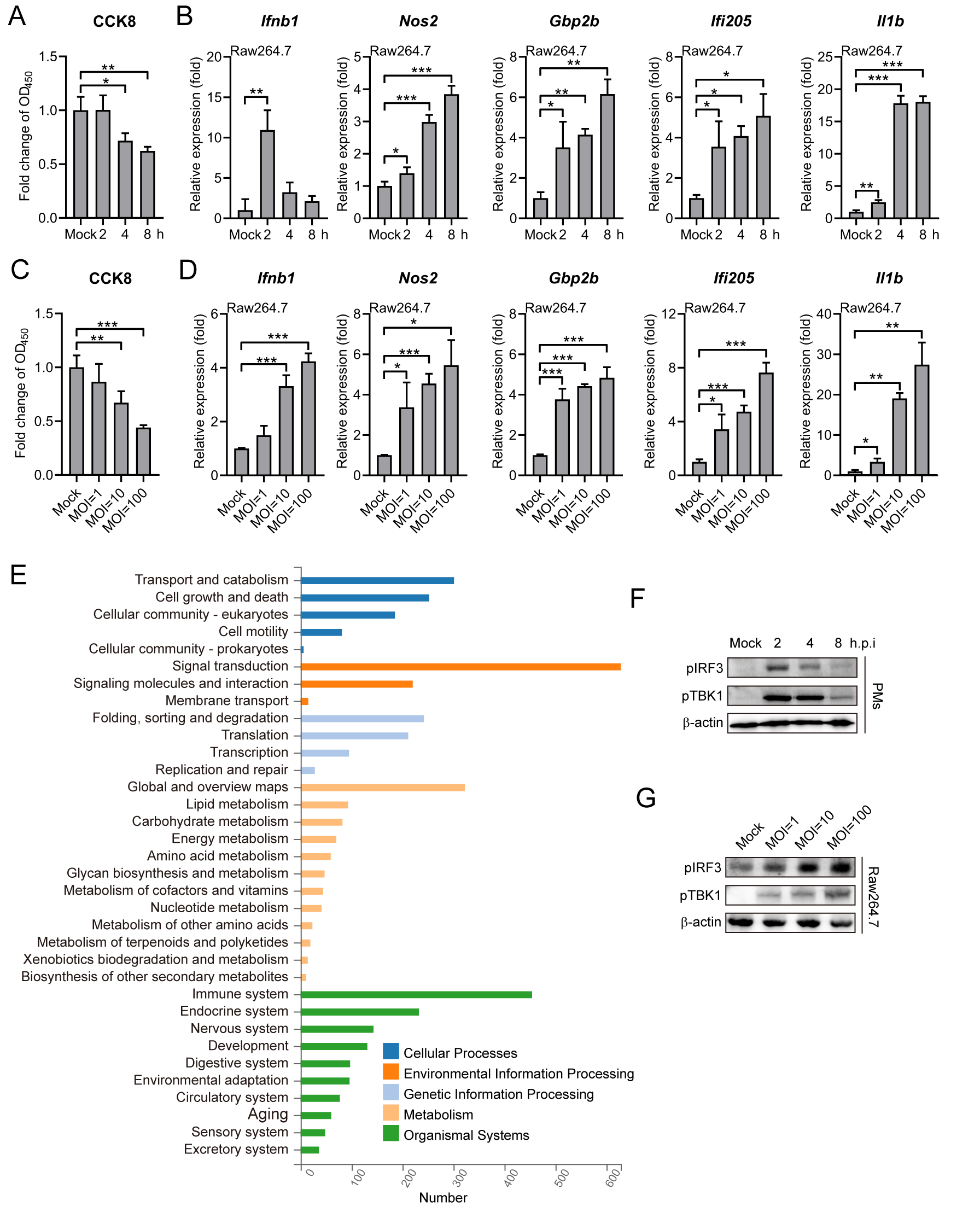

Supplement: FIG S1 [file mbio.03632-21-sf001.tif]

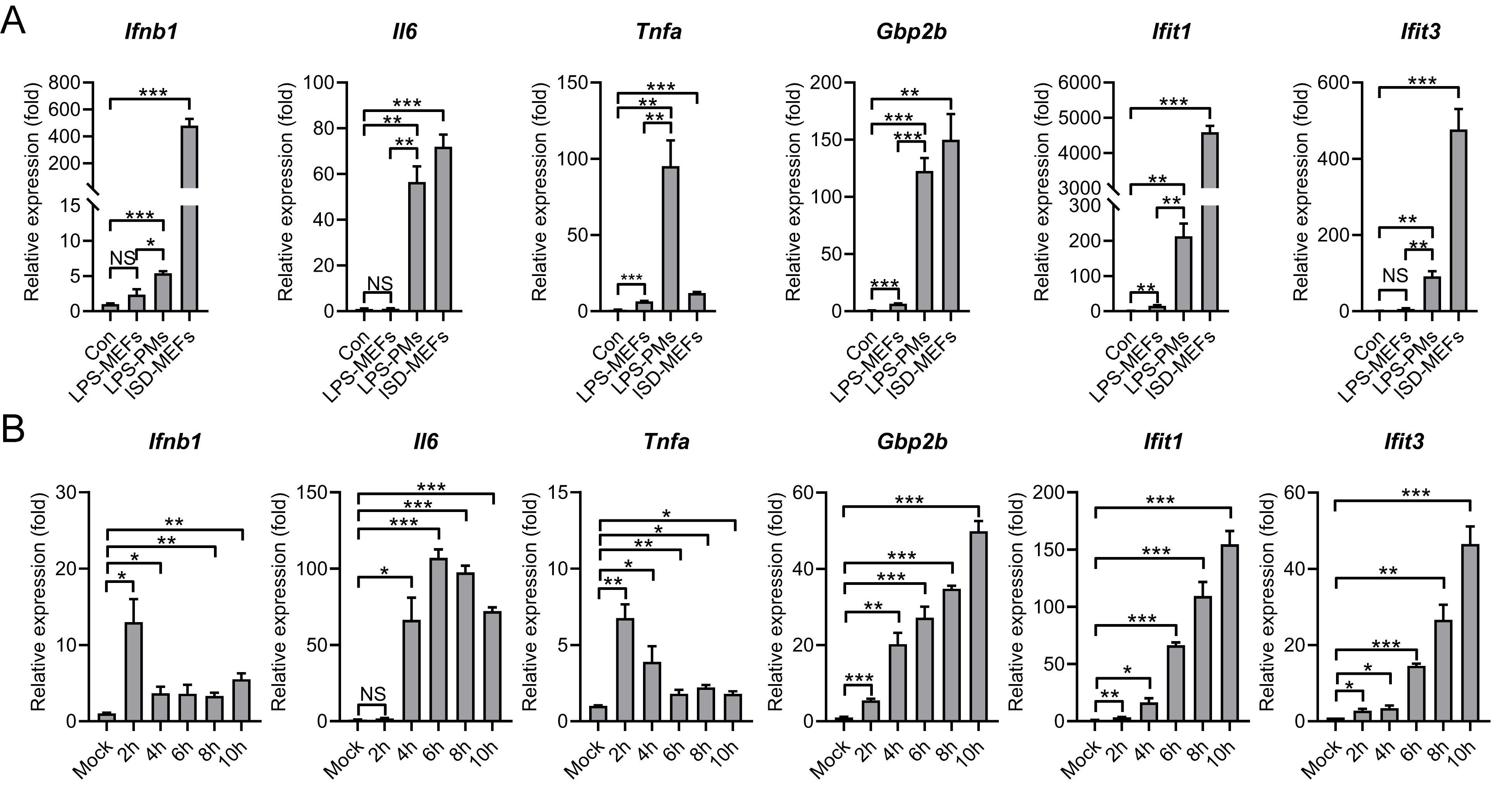

Supplement: FIG S2 [file mbio.03632-21-sf002.tif]

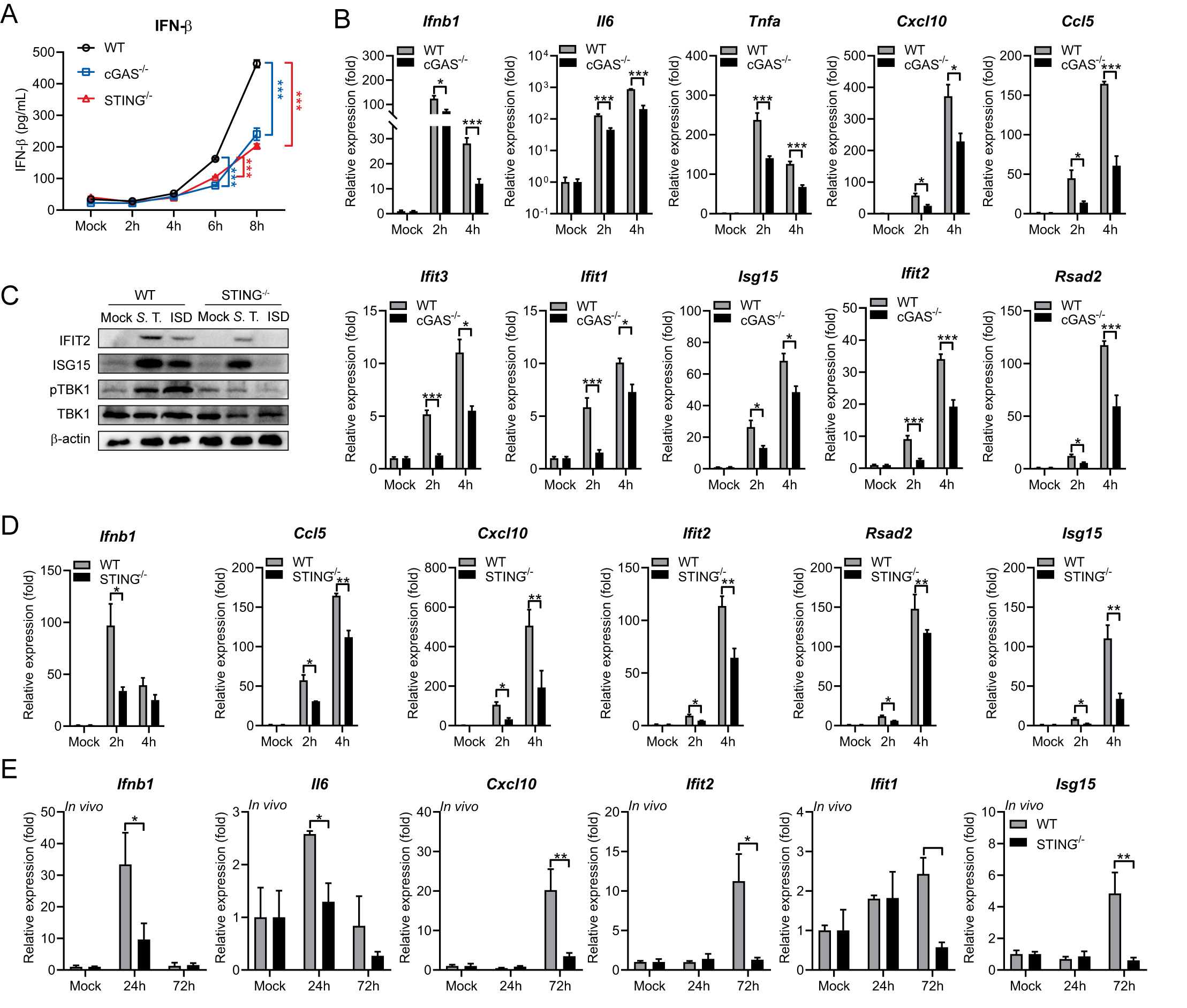

Supplement: FIG S3 [file mbio.03632-21-sf003.tif]

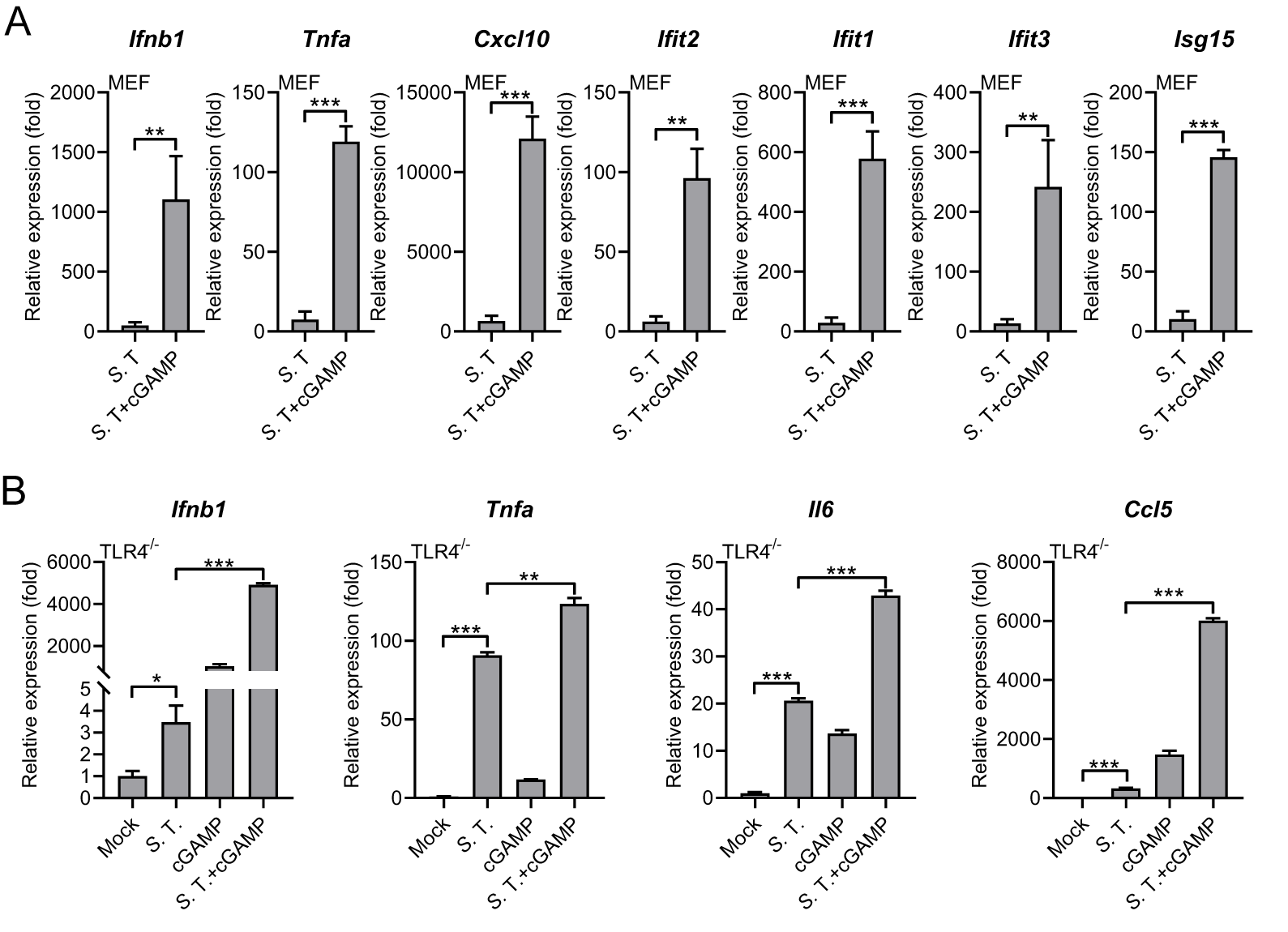

Supplement: FIG S4 [file mbio.03632-21-sf004.tif]

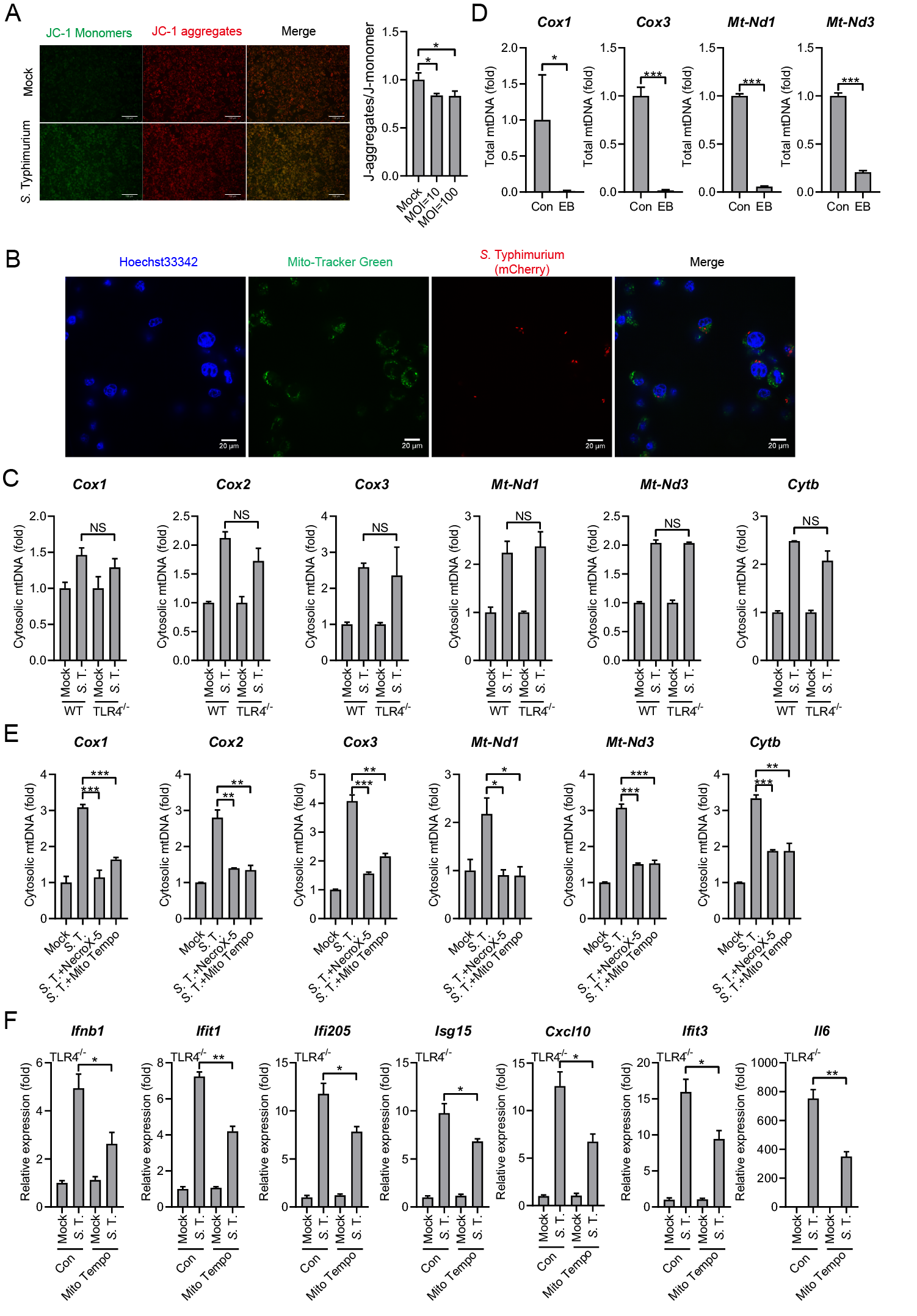

Supplement: FIG S5 [file mbio.03632-21-sf005.tif]

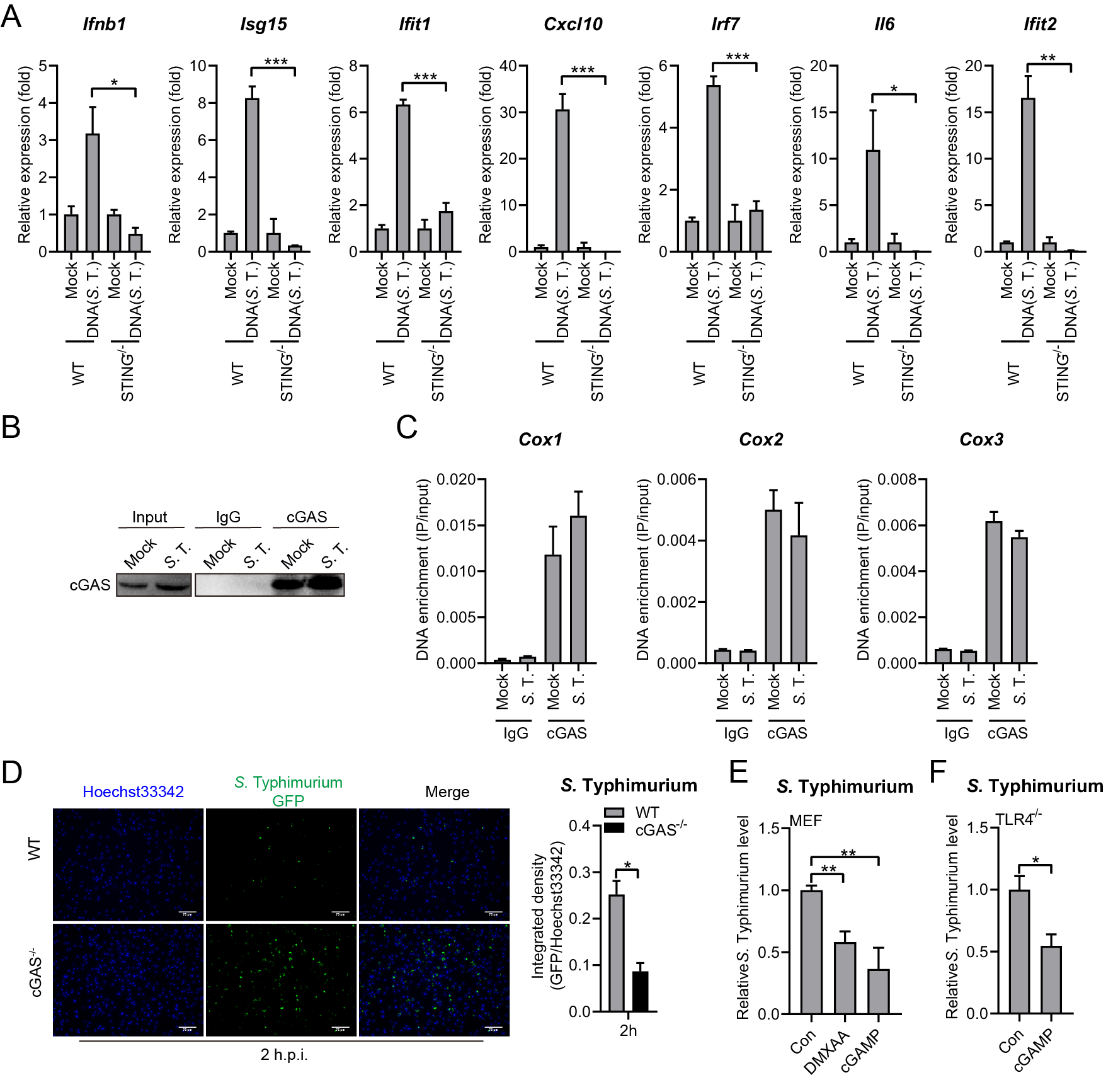

Supplement: FIG S6 [file mbio.03632-21-sf006.tif]

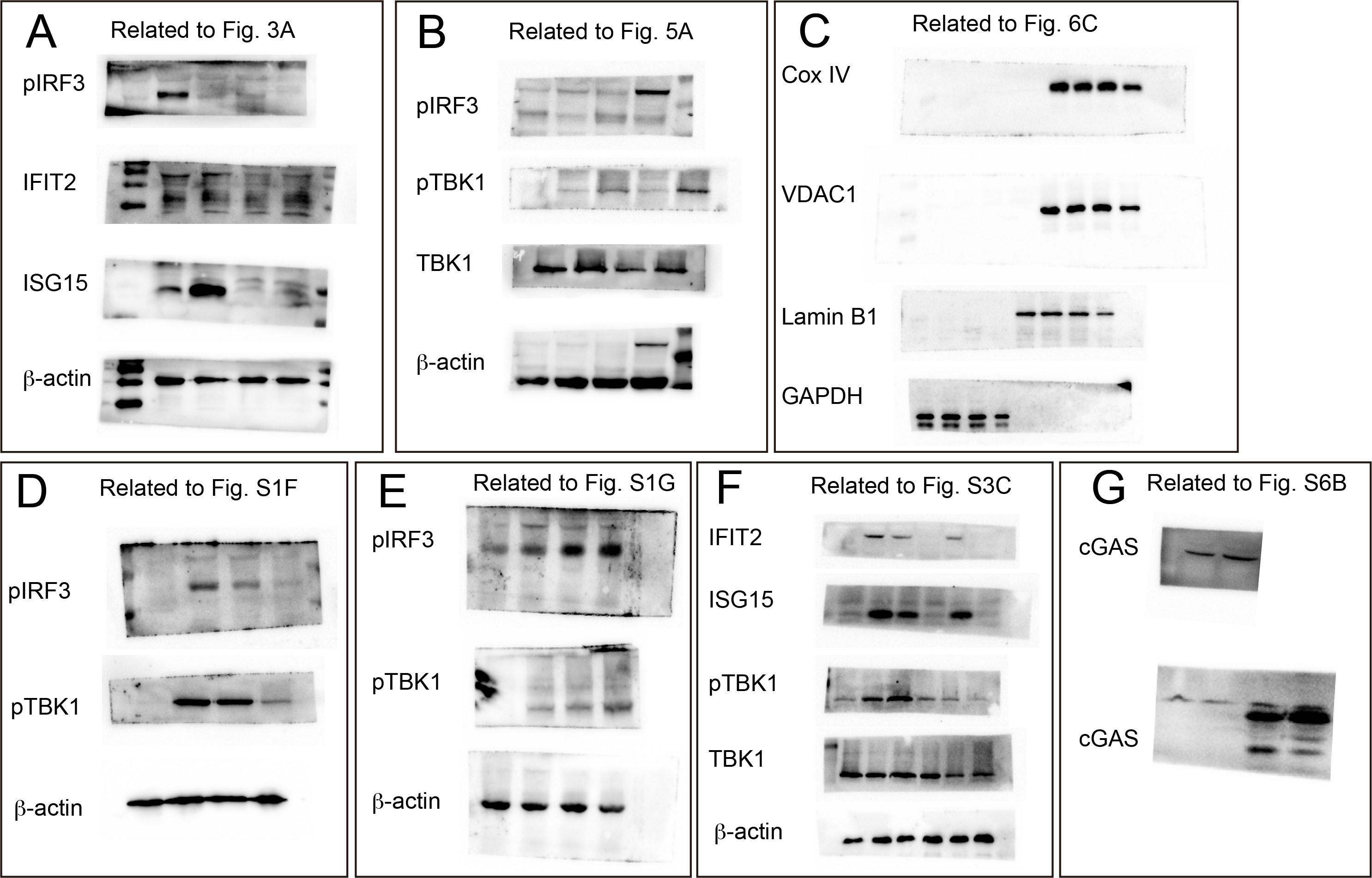

Supplement: FIG S7 [file mbio.03632-21-sf007.jpg]
