# Supplementary material for: Salmonella Induces the cGAS-STING-Dependent Type I Interferon Response in Murine Macrophages by Triggering mtDNA Release
Source: mBio. 2022 May 23;13(3):e03632-21. doi: 10.1128/mbio.03632-21 (PMC9239183; doi:10.1128/mbio.03632-21)
Supplement: TEXT S1 [file mbio.03632-21-s0001.docx]

# Supplementary Materials and Methods

## Bacterial strains

The *S*. Typhimurium used in this study was the *Salmonella enterica* serovar Typhimurium SL1344 strain, which has a streptomycin resistance ability. Genes encoding GFP or mCherry were inserted into the pKT100 plasmid and electro-transformed into *S*. Typhimurium. All of these strains were cultured in Luria Bertani (LB) with appropriate antibiotics at 37°C. Cellular growths were monitored based on the optical density OD at 600 nm (OD_600_). Antibiotics were added at the following concentrations: streptomycin and kanamycin at 100 μg/mL or 50 μg/mL, respectively. All Bacterial strains were listed in Table S2.

## Primary cells and cell lines

Raw264.7, HeLa and primary mouse embryonic fibroblast (MEF) cells used in this study were cultured in DMEM (High-glucose), supplemented with 10% FBS, penicillin (100 U/mL) and streptomycin (100 μg/mL). WT HeLa cells were kindly provided by Dr. Zhengfan Jiang ([1](#_ENREF_1)). Primary murine macrophages were prepared as previously described ([2](#_ENREF_2)). Mouse peritoneal macrophages (PMs) were harvested from mice after intraperitoneal injection with beef extract peptone medium (0.3% beef extract, 1% peptone, 0.5% NaCl and 6% Soluble starch) for 3 days, and were cultured in RPMI 1640 medium, supplemented with 10% FBS, penicillin (100 U/mL), streptomycin (100 μg/mL), 10 μM sodium pyruvate, 0.1 mM non-essential amino acids, 50 mM 2-mercaptoethanol and 25 mM HEPES for 1 day. Primary MEFs were generated from E12.5–14.5 embryos as described and sub-cultured no more than five passages before experiments ([3](#_ENREF_3), [4](#_ENREF_4)).

## RNA isolation and quantitative real-time PCR

RNAeasy Animal RNA Isolation Kit with Spin Column (Beyotime Biotechnology, #R0027) was used to isolate RNA from mammalian cells. For the detection of intracellular *S*. Typhimurium RNA level, infected cells were lysed by Trizol Regnant (Sigma-Aldrich, #T9424) and total RNA was extracted by using chloroform and isopropanol according to the manufacturer’s instructions. The purity and concentration of the RNA were determined by gel electrophoresis and spectrophotometer (NanoDrop, Thermo Scientific). RNAs were reversed with random primer by Reverse Transcription kit (Transgene, #AH311-02) and quantified with special primers by SYBR FAST qPCR Kit (KAPA Biosystem, #KK4601). The cycling proceeding was used as follows: 95°C for 150 s followed by 45 cycles of 94°C for 10 s, 52°C or 58°C for 30 s. Quantitative real-time PCR (qRT-PCR) was performed in LightCycler 96 System (Roche). The relative abundance of *S*. Typhimurium *16S* rRNA was used to indicate the level of intracellular *S*. Typhimurium RNA level. *Actin* was used as the internal standard of mammalian cells. All samples were analyzed in triplicate, and the expression of target genes was calculated as relative fold values using the 2^-ΔΔCt^ method. All primers for qRT-PCR are listed in Table S2.

## RNA-Seq experiment

Whole transcriptome sequencing was performed at BGI-Shenzhen (Shenzhen, China). PMs from C57BL/6 mice were infected by *S*. Typhimurium strains at an MOI of 10 for 30 min. Then the cells were cultured in RPMI 1640 supplemented with 200 μg/mL Gentamicin after washing by PBS two times and subsequently incubated at 37°C with 5% CO_2_ for 8 h. Total RNA was extracted from the cells using TRIzol (Sigma-Aldrich, #T9424) and RNA degradation and contamination were monitored on 1% agarose gels and RNA purity was checked using the NanoPhotometer spectrophotometer (Implen) and RNA integrity was assessed using the Bio-analyzer 2100 system. The sequencing proceeds were performed as described before ([2](#_ENREF_2)). Oligo(dT)-attached magnetic beads were used to purify mRNA. cDNA synthesis, end repair, A-base addition, and ligation of the Illumina-indexed adaptors were performed according to the instructions. The double-stranded PCR products from the previous step were heat-denatured and circularized by the splint oligo sequence to get the final library. The single-strand circle DNA (ssCir DNA) was formatted as the final library. The final library was amplified with phi29 to make the DNA nanoball (DNB). DNBs were loaded into the patterned nanoarray and single end 50 bases reads were generated on BGIseq500 platform. DESeq2 was used for the differential expression analysis and a threshold was set to a Q value with a false discovery rate (FDR) < 0.05 and a fold change cutoff > 2. Raw FASTQ files for the RNA-seq libraries are deposited in the NCBI Sequence Read Archive (SRA) and have been assigned BioProject accession PRJNA764332.

## Western blot analysis

Proteins in samples were resolved by SDS-PAGE and transferred onto PVDF membranes (Millipore). The membrane was blocked with QuickBlock Blocking Buffer (Beyotime Biotechnology, #P0252) for 2 h at 4°C and incubated with primary antibodies at 4°C overnight. The membrane was washed three times in TBST buffer (50 mM Tris, 150 mM NaCl, 0.05% Tween 20, pH 7.4), and incubated with horseradish peroxidase-conjugated secondary antibodies for 4 h at 4°C. Signals were detected using the ECL kit (Invitrogen) following the manufacturer's specified protocol. All antibodies are listed in Table S2.

## Subcellular fractionation extraction

The way of extraction of cytosolic fractionation was adapted from Aarreberg ([5](#_ENREF_5)). Approximately 10^6^ Raw264.7 cells were lysed in 500 μL Digitonin buffer (150 mM NaCl, 50 mM HEPES pH 7.4, 40 μg/mL Digitonin, Protease and phosphatase inhibitors) on a shaker at 4°C for 10 minutes. Cells were collected and centrifuged at 2000*×g* for 10 min at 4°C. Supernatants were transferred to fresh tubes and centrifuged at 20000*×g* for 20 min at 4°C and this step was repeated 3 times and the supernatants were cytosolic fractions. Split the sample into two tubes, one for mitochondrial DNA detection and one for immunoblotting. The remaining pellet from the first spin was re-suspended in PBS and split into two new tubes, one for total DNA extraction and one for immunoblotting. DNA was extracted from the nuclear fractions using the NucleoSpin DNA RapidLyse (Macherey-Nagel). Cytosolic fractions were diluted into DNase-free water at 1:20 and qPCR was used to quantify mitochondrial DNA. Nuclear gene *Actin* was quantified from the nuclear fraction for normalization.

## Mitochondrial depletion

Raw264.7 cells were cultured in DMEM with or without 150 ng/mL Ethidium Bromide (EB) for 4 days ([5](#_ENREF_5), [6](#_ENREF_6)). On Day 4, cells were washed with PBS trypsinized and seeded in the 24-well plate at a density of 2 × 10^5^ per well in DMEM without EB. Cells were infected with *S*. Typhimurium on the second day after being attached to the plate bottom. Total RNA was collected by RNAeasy Animal RNA Isolation Kit with Spin Column (Beyotime Biotechnology, #R0027). Total DNA from cells with or without EB treatment was extracted and quantified with special mitochondrial primers to ensure the depletion.

## Measurement of mitochondrial membrane potential

The Mitochondrial membrane potential assay kit with JC-1 (Beyotime Biotechnology, #C2006) or TMRE (Beyotime Biotechnology, #C2001S) was used to measure mitochondrial membrane potential according to the manufacturer’s instructions. HeLa cells were seeded in the 96-well flat-bottom clear plate (Corning, #CLS3615) with a density of 2×10^4^ per well. The next day, cells were infected with *S*. Typhimurium at an MOI at 100 for 2 h. Infection media was removed and replaced with DMEM containing FBS (10%) and gentamicin (200 μg/mL) for indicated time to kill extracellular bacteria. JC-1 working solution was added to cells at 1:1 of the volume of the culture medium and incubated for 20 min. Then, cells were washed twice with precooled JC-1 buffer (supplied in the kit). The cells were imaged by using an inverted fluorescence microscope (DMi8, Leica) and fluorescence spectrophotometer (Spectra Max M 2, Molecular Devices) to detect JC-1 monomers fluorescence at 490-530 nm and JC-1 aggregates fluorescence at 525-590 nm. The result was presented with monomers/aggregates to show the change of mitochondrial membrane potential. As for TMRE, Raw264.7 cells were seeded in the confocal dish (Beyotime Biotechnology, #FCFC016-10pcs) with a density of 1×10^5^ per dish. TMRE was diluted by assay buffer (supplied in the kit) at a ratio of 1:1000 and incubated cells for 15 min. The cells were washed twice with prewarmed PBS and imaged using a confocal laser scanning microscopy (STELLARIS 8 FALCON, Leica) to detect TMRE fluorescence at 550-575 nm. Analyze the fluorescence density of infected and uninfected cells.

## Co-immunoprecipitation of cGAS and DNA analysis

Co-immunoprecipitation of cGAS and DNA analysis were performed as previously described ([7](#_ENREF_7), [8](#_ENREF_8)). Briefly, RAW267.4 cells were infected with *S*. Typhimurium, washed with cold PBS and fixed in 4% PFA. Next, cells were lysed using lysis buffer. Samples were then immunoprecipitated with protein A/G Magnetic Beads pre-cross-linked with cGAS antibody. Whole-cell lysates (Input) or IPs were visualized by Western blot using an anti-cGAS antibody. Samples were de-crosslinked at 65°C for 16 h and then treated with Proteinase K. DNA was isolated and qPCR was then performed to measure the abundance of fragments from *S*. Typhimurium. All primers are listed in Table S2.

## Plasmids and construction

Plasmid pKT100 was inserted with *gfp* or *mCherry* gene to obtain pKT100-*gfp* and pKT100-*mCherry* constructs ([2](#_ENREF_2)). The PCR products of *gfp* or *mCherry* gene were digested with BamH I/Sal I and inserted into the BamH I/Sal I sites of pKT100 to obtain pKT100-*gfp* and pKT100-*mCherry* constructs. The integrity of the inserts in all constructs was confirmed by DNA sequencing. All primers for plasmid construction are listed in Table S2.

## Isolation and transfection of DNA or cGAMP

To isolate DNA from *S.* Typhimurium, bacteria were cultured in LB at 37°C overnight and the bacterial pellet was collected. DNA was extracted by using NucleoSpin DNA RapidLyse (Macherey-Nagel). Transfection was achieved by Lipofectamine 3000 Transfection Reagent (Invitrogen, #L3000008). The cells were seeded in 24-well plates the day before and were transfected at the density of 60% with interferon stimulatory DNA (ISD, 45 bp dsDNA), *S.* Typhimurium genomic DNA or 2’3’-cGAMP. At 6 h post transfections, samples were collected to detect gene expression or cells were used for subsequent infection.

## ELISA

IFN-β production was detected by Mouse Interferon β, IFN-β/IFNB ELISA Kit (CUSABIO, #CSB-E04945m) according to the manufacturer’s instructions. Mouse PMs were seeded in the 24-well plates and infected with *S*. Typhimurium at an MOI of 10. Culture supernatants from indicated h.p.i. were collected to detect the production of IFN-β. Precoated ELISA plates were incubated with 100 µL samples at 37°C for 2 h, then discard liquid and add 100 µL antibody solution, incubate at 37°C for 1 h. Aspirate and wash wells 3 times with wash buffer (supplied in the kit). Incubate wells with 100 µL HRP Solution at 37°C for 1 h. Aspirate and wash 5 times. Add 90 µL of TMB Substrate to each well and incubate for 10 min at 37°C in the dark. Finally, add 50 μL of Stop Solution and read the absorbance at 450 nm within 5 minutes.

## Cell toxicity test

Cell toxicity assays were achieved according to the manufacturer’s instructions of Cell Counting Kit 8 (CCK8) (Mishushengwu, #MI00612). Mouse PMs were seeded in 96-well plates and infected with *S.* Typhimurium at indicated timepoints and MOIs. The cells were stained with regnant in the kits with a concentration of 10% (v/v) for 30 min and the absorbance at 450 nm (OD_450_) was measured with a microplate reader.

## Statistical analysis

Experimental data analyzed for significance were performed by using GraphPad Prism 6 (GraphPad Software, San Diego, California, USA). *P* values for mice survival were calculated using the Log-rank (Mantel-Cox) test. *P* values for bacterial CFU in mouse tissues were calculated using the Mann-Whitney test (I). Statistical analyses for the rest of the assays were performed using paired two-tailed Student’s t-test. Error bars represent ± SEM. **P*< 0.05; ***P*< 0.01; ****P*< 0.001.

# References

1. Wang C, Guan Y, Lv M, Zhang R, Guo Z, Wei X, Du X, Yang J, Li T, Wan Y, Su X, Huang X, Jiang Z. 2018. Manganese increases the sensitivity of the cGAS-STING pathway for double-stranded DNA and is required for the host defense against DNA viruses. Immunity 48:675-687 e7.

2. Zhu L, Xu L, Wang C, Li C, Li M, Liu Q, Wang X, Yang W, Pan D, Hu L, Yang Y, Lu Z, Wang Y, Zhou D, Jiang Z, Shen X. 2021. T6SS translocates a micropeptide to suppress STING-mediated innate immunity by sequestering manganese. Proc Natl Acad Sci U S A 118.

3. Durkin ME, Qian X, Popescu NC, Lowy DR. 2013. Isolation of Mouse Embryo Fibroblasts. Bio Protoc 3.

4. Qiu LQ, Lai WS, Stumpo DJ, Blackshear PJ. 2016. Mouse Embryonic Fibroblast Cell Culture and Stimulation. Bio Protoc 6.

5. Aarreberg LD, Esser-Nobis K, Driscoll C, Shuvarikov A, Roby JA, Gale M, Jr. 2019. Interleukin-1beta Induces mtDNA Release to Activate Innate Immune Signaling via cGAS-STING. Mol Cell 74:801-815 e6.

6. Hashiguchi K, Zhang-Akiyama QM. 2009. Establishment of human cell lines lacking mitochondrial DNA. Methods Mol Biol 554:383-91.

7. Zhou CM, Wang B, Wu Q, Lin P, Qin SG, Pu QQ, Yu XJ, Wu M. 2021. Identification of cGAS as an innate immune sensor of extracellular bacterium *Pseudomonas aeruginosa*. iScience 24:101928.

8. Watson RO, Bell SL, MacDuff DA, Kimmey JM, Diner EJ, Olivas J, Vance RE, Stallings CL, Virgin HW, Cox JS. 2015. The Cytosolic Sensor cGAS Detects *Mycobacterium tuberculosis* DNA to Induce Type I Interferons and Activate Autophagy. Cell Host Microbe 17:811-819.
